# A population genetic window into the past and future of the walleye *Sander vitreus*: relation to historic walleye and the extinct “blue pike” *S. v. “glaucus”*

**DOI:** 10.1186/1471-2148-14-133

**Published:** 2014-06-17

**Authors:** Amanda E Haponski, Carol A Stepien

**Affiliations:** 1 The Great Lakes Genetics/Genomics Laboratory, Lake Erie Center and Department of Environmental Sciences, The University of Toledo, 6200 Bayshore Road, Toledo, OH 43616, USA

**Keywords:** Biogeography, Blue pike, Control region, Microsatellites, *Sander*, Walleye

## Abstract

**Background:**

Conserving genetic diversity and local adaptations are management priorities for wild populations of exploited species, which increasingly are subject to climate change, habitat loss, and pollution. These constitute growing concerns for the walleye *Sander vitreus,* an ecologically and economically valuable North American temperate fish with large Laurentian Great Lakes' fisheries. This study compares genetic diversity and divergence patterns across its widespread native range using mitochondrial (mt) DNA control region sequences and nine nuclear DNA microsatellite (μsat) loci, examining historic and contemporary influences. We analyze the genetic and morphological characters of a putative endemic variant– “blue pike” *S. v. “glaucus”* –described from Lakes Erie and Ontario, which became extinct. Walleye with turquoise-colored mucus also are evaluated, since some have questioned whether these are related to the “blue pike”.

**Results:**

Walleye populations are distinguished by considerable genetic divergence (mean *F*_ST_ mtDNA = 0.32 ± 0.01, μsat = 0.13 ± 0.00) and substantial diversity across their range (mean heterozygosity mtDNA = 0.53 ± 0.02, μsat = 0.68 ± 0.03). Southern populations markedly differ, possessing unique haplotypes and alleles, especially the Ohio/New River population that houses the oldest haplotype and has the most pronounced divergence. Northern formerly glaciated populations have greatest diversity in Lake Erie (mean heterozygosity mtDNA = 0.79 ± 0.00, μsat = 0.72 ± 0.01). Genetic diversity was much less in the historic Lake Erie samples from 1923–1949 (mean heterozygosity mtDNA = 0.05 ± 0.01, μsat = 0.47 ± 0.06) than today. The historic “blue pike” had no unique haplotypes/alleles and there is no evidence that it comprised a separate taxon from walleye. Turquoise mucus walleye also show no genetic differentiation from other sympatric walleye and no correspondence to the “blue pike”.

**Conclusions:**

Contemporary walleye populations possess high levels of genetic diversity and divergence, despite habitat degradation and exploitation. Genetic and previously published tagging data indicate that natal homing and spawning site philopatry led to population structure. Population patterns were shaped by climate change and drainage connections, with northern ones tracing to post-glacial recolonization. Southerly populations possess unique alleles and may provide an important genetic reservoir. Allelic frequencies of Lake Erie walleye from ~70–90 years ago significantly differed from those today, suggesting population recovery after extensive habitat loss, pollution, and exploitation. The historic “blue pike” is indistinguishable from walleye, indicating that taxonomic designation is not warranted.

## Background

Species today face many challenges that influence their genetic, phenotypic, and ecological diversity and divergence patterns across space and time. The genetic variation of their component populations comprises the raw material underlying overall and local adaptedness, providing resilience to anthropogenic stressors –such as climate change, habitat alteration and loss, invasive species, and exploitation [1,2]. Notably, declines in genetic diversity may lead to decreased fitness, undermining ability to respond to present and future conditions [3,4]. Evaluating comparative and hierarchical levels of genetic composition and differentiation of widespread taxa, as well as their unique or reservoir populations, thus is important for prioritizing conservation management strategies [5,6].

Population genetic patterns of today’s temperate taxa resulted from historic and contemporary processes [7,8], regulated by their physiological requirements, habitat connectivity, and dispersal capability [9,10]. Broadly distributed taxa display heterogeneous patterns over a breadth of environmental and ecological conditions [11,12]. Isolated populations experience drift and possibly evolve unique alleles [13,14], whereas large inter-connected ones frequently have high gene flow and less genetic distinctiveness [15,16]. Low migration may lead to higher divergences, whereas mobility fosters gene flow and homogeneity [17,18].

**Figure 1:**
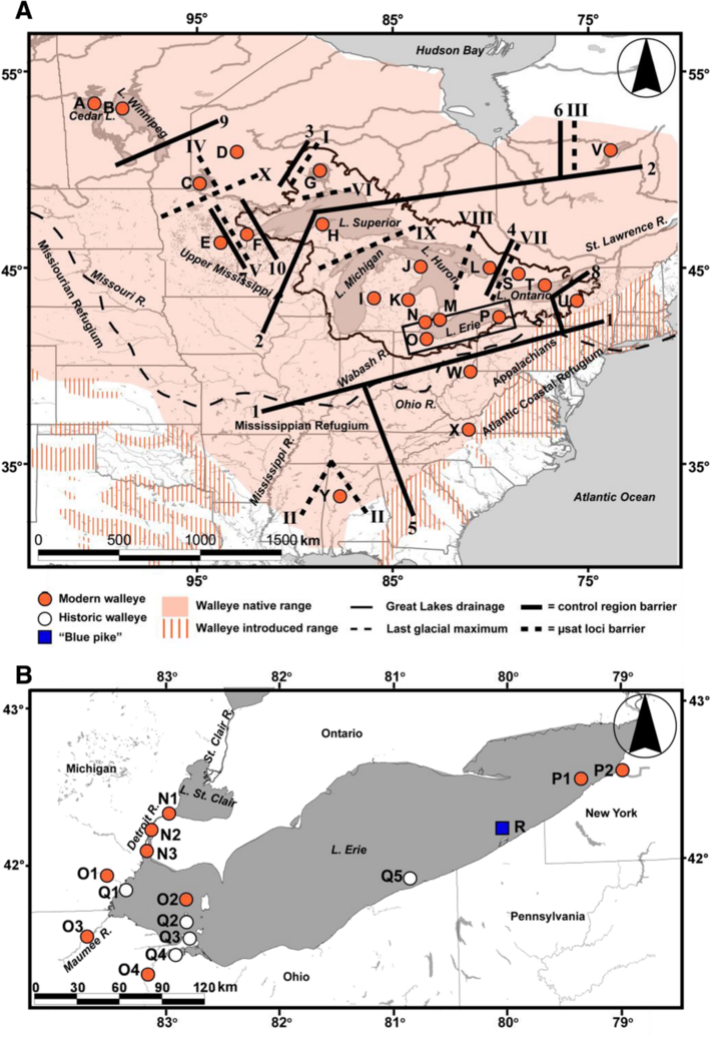
**Maps of walleye sampling sites (lettered, corresponding to locations in Table** 1**and Additional file**10**). A** Distribution across North America (modified from Billington et al. [25]), depicting genetic discontinuities among contemporary walleye spawning groups from Barrier v2.2 analysis [26], with black box denoting area of close-up study for **B** Lake Erie, with historic collections of walleye (labeled Q) and “blue pike” (R). Maps were created by us with ArcGIS® software by Esri (Redlands, CA).

During the Pleistocene Epoch, ~2.6–0.01 million years ago, the North American Laurentide Ice Sheet advanced south to the Ohio River system (Figure 1), altering population distributions and genetic compositions [19,20]. Taxa became sequestered in glacial refugia and then moved northwards to colonize old and new habitats as the ice retreated [21,22]. Recent climate warming is accelerating these historic dispersal patterns northward [23,24].

Anthropogenic factors may further modify genetic patterns and force populations into sub-optimal habitats leading to reduced population size, genetic diversity, and/or local adaptation and resulting in possible extirpation [27]. For example, increasing temperatures may reduce population sizes and ranges of temperate cold-water fishes, e.g., lake trout *Salvelinus namaycush* (Walbaum 1792) and cutthroat trout *Oncorhynchus clarkii* (Richardson 1836) [23,28]. Rising temperatures and decreasing population sizes have been linked to declining genetic diversity through drift and inbreeding in Atlantic salmon *Salmo salar* Linnaeus 1758 and brown trout *S. trutta* Linnaeus 1758 [29]. In contrast, warm-water species, such as the walleye *Sander vitreus* (Mitchill 1818) may broaden their ranges, shifting their distribution centers north [23]. Some outlying populations may fail to adapt, leading to declining genetic variability and extirpation [28], which may particularly affect southerly fringe areas such as the relict North River, Alabama population of walleye [30] (site Y; Figure 1). Exploitation and other anthropogenic stressors may accelerate diversity loss further [2,31]. The present study analyzes the genetic patterns of walleye, in light of its past and potential future, to evaluate such patterns of variability, isolation, and continuity over space and time.

The walleye is an ecologically and economically valuable fish [32,33] that supports large Great Lakes’ fisheries, peaking in Lake Erie [34]. It inhabits slow turbid lakes to fast flowing clear streams throughout much of North America (Figure 1). Historically, it ranged from the Mackenzie River in the Northwest Territories of Canada, south to the U.S. Gulf Coast, and northeastward to New Hampshire and Quebec; during the past century it also was transplanted into many other areas for fishing [25] (Figure 1A). After maturing at ~ age three, it migrates annually in spring to early summer to reproduce at natal spawning grounds [35], exhibiting site fidelity, homing, and philopatry [36]. Adults do not provide parental care or nest guarding, and range widely to feed after spawning, travelling 50–300 km [37].

Past studies by our laboratory examined fine-scale population patterns of walleye using nine nuclear DNA microsatellite (μsat) loci, showing that most spawning groups markedly differ even within lake basins and between proximate sites, as well as at broad scales supporting natal homing and spawning site philopatry [30,38-40]. Stepien et al. [38] found that the genetic structure of spawning groups remains similar from year to year, among age cohorts, and from generation to generation. An earlier study [41] addressed patterns across the Great Lakes using mitochondrial (mt) DNA control region sequences, whose geographic scope is extended here to accompany the μsat data set and includes new information from the Canadian Shield lakes for both data sets.

We additionally evaluate the historic genetic diversity of walleye from Lake Erie using preserved samples from 1923–1949. We address the taxonomic identity of a possible historic walleye variant, the “blue pike” *S. v. “glaucus”* (Hubbs [42]), whose distinction has been controversial [41,43]. The “blue pike” was reported to be endemic in Lakes Erie and Ontario, where it co-occurred with the common walleye *S. v. vitreus* (hereafter referred to as walleye). The “blue pike” was believed to inhabit the deeper cooler waters of eastern Lake Erie, but also was caught in the shallow and warmer western basin along with walleye [44]. It reportedly spawned somewhat later and in deeper areas than walleye [43]. The “blue pike” was described to have a steel grey-blue color, larger eyes that were higher on the head, and a smaller interorbital distance than walleye [44,45]. However, all characters overlapped extensively between the two [44]. Hubbs [42] originally described the “blue pike” as a separate species (“*S. glaucus*”), which then was demoted to a subspecies due to pronounced intergrades with walleye including color and all other morphological measures [44]. The present study thus addresses their identities using new morphological and genetic data.

Both “blue pike” and walleye shared a popular commercial fishery with the former collapsing in 1959 attributed to exploitation, pollution, and/or habitat alteration and the latter concurrently declining [44]. The last “blue pike” record was reported in 1965 [44] and the US Fish and Wildlife Service declared it officially extinct in 1983 [46]. The “blue pike’s” identity has been confusing since it did not belong to the pike family (Esocidae) and some walleye in northern waters (along the Canadian Shield) are colored bright turquoise-blue due to sandercyanin protein in the mucus [47], which does not match the darker grey-blue color described for “blue pike” [48,49]. Early fishery biologists called these turquoise mucus walleye “mutants” and stated that they lacked the body color, shape, and other morphological characteristics of “blue pike” [49]; however, these variant colors have become confused in the literature [41]. Turquoise mucus and yellow walleye occur sympatrically in the same northern habitats, and the latter also have some turquoise mucus pigment [45]. The turquoise mucus additionally characterizes some yellow perch *Perca flavescens* (Mitchill 1814) in those habitats. Some of the turquoise color typically “rubs off” when the fish is collected [46 and CAS, pers. obs.].

This study aims to resolve population genetic patterns of walleye across its native range, providing a baseline for evaluating future anthropogenic pressures. We analyze contemporary walleye spawning groups and compare Lake Erie populations to historic samples, including the putative “blue pike” variant. Morphological characters also are analyzed for the historic samples. We expand the previous sampling coverage to 1181 walleye and combine information from mtDNA control region sequences and nine nuclear DNA μsat loci. Patterns are evaluated at multiple evolutionary and temporal scales, with mtDNA sequences revealing historical context (glacial refugium origins and taxonomic relationships) and μsat loci addressing contemporary microevolutionary processes (migration, gene flow, and genetic drift) [50,51]. Biogeographic patterns are compared with those of other North American taxa*.* Specific questions are: (1) What is the genetic structure of walleye across its native distribution?, (2) How does genetic diversity vary across the range?, (3) What is the relationship of contemporary samples to historic Lake Erie (1923–1949) patterns?, and (4) Did the extinct “blue pike” significantly differ from walleye?

## Results

### Genetic diversity and phylogenetic relationships from mtDNA

**Figure 2:**
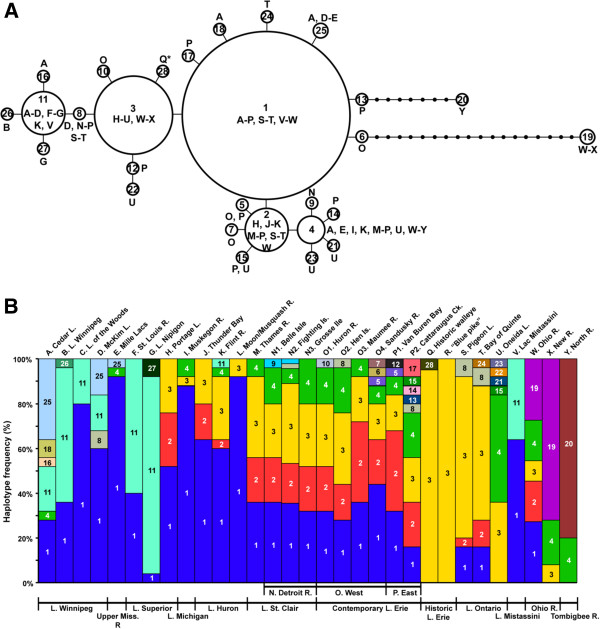
**Relationships among mtDNA control region haplotypes of walleye, including A haplotype network from TCS v1.21 [**[54]**] and B relative frequencies per population sample.** Haplotypes from contemporary spawning groups are numbered 1–27 and #28 was found in a historic walleye**.** Letters = geographic locations of haplotypes from Table 1. * = haplotypes occurring in the historic walleye and “blue pike” samples analyzed here. Circles in the network are sized according to total observed frequency of the haplotype. Lines = a single mutational step between the haplotypes; small unlabeled circles = those hypothesized/not sampled.

Contemporary walleye populations contain 27 control region haplotypes (Figure 2; GenBank Accession numbers U90617, JX442946–56, KC819843–54, KF954732–35). Haplotypes 1–23 match those previously described by our laboratory [41,52,53] and five additional ones are identified here (four from contemporary samples, haplotypes 24–27; GenBank:KF954732–35, and one from historic Lake Erie walleye, haplotype 28; GenBank:KF954736) –totaling 28 haplotypes. Haplotype 19 from the Ohio/New Rivers (W–X, 1.00pp/98%) is located basally on the phylogenetic tree as the sister clade to all other walleye haplotypes, diverging by 19 steps (sites W and X; Additional file 1). The remaining 27 haplotypes share a common origin, with most differing by just single mutational steps, excepting haplotype 20 from the North River (site Y), which differs by eight (Figure 2A). Historic walleye haplotype 28 groups with the others (Additional file 1), varying by just a single mutational step from abundant haplotype 3 and found in a single individual.

Five abundant haplotypes (1–4, 11) comprise 90% of walleye individuals, with haplotype 1 being the most frequent and widely distributed (Figure 2B, Additional file 2). Haplotype 3 is the next most numerous, and is distributed from the upper Great Lakes through the south. It is the sole haplotype shared between the contemporary and historic samples; it predominated in historic Lake Erie walleye and characterized all “blue pike” samples, in contrast to being represented in just 22% of today's Lake Erie walleye (Figure 2B, Additional file 2). The historic “blue pike” samples thus do not genetically differ from historic and contemporary walleye in mtDNA control region sequences. Haplotypes 2 and 4 are less abundant, yet have wide distributions and characterize a large number of individuals and locations. Haplotype 11 is broadly distributed and abundant in the northern samples (A–G, K, V), but mostly is absent from other areas (Figure 2B, Additional file 2). Three other haplotypes appear unique to separate drainages: haplotype 25 in Lake Winnipeg and the Upper Mississippi River (A, D–E), haplotype 19 from the Ohio/New Rivers (W–X), and haplotype 20 in the North River (Y).

Each spawning group contains multiple haplotypes (Figure 2, Table 1), with Lake Erie having the most (O–P) and Lake of the Woods (C), St. Louis River (F), Moon/Musquash Rivers (L), Lac Mistassini (V), and North River (Y) having the least. Historic walleye and “blue pike” appear to have possessed fewer haplotypes compared to populations in Lakes Erie and Ontario today. Modest numbers and proportions of private haplotypes characterize contemporary populations, with Oneida Lake (U) and North River (Y) containing the most. Lake Erie walleye have more private haplotypes than other Great Lakes' locations. A rare private haplotype (28) occurred in a single historic walleye individual, which appears to have been lost to today's populations. Historic “blue pike” samples lacked private haplotypes.

**Table 1:** Genetic variation of contemporary walleye populations (sampling areas labeled A–P, S–Y), historic walleye (Q), and “blue pike” (R) determined from mtDNA control region sequence data and nuclear DNA μsat loci

	**Control region**					**μsat loci**						
**Location**	***N***	***H***_**D**_**±S.E.**	***N***_**H**_	***N***_**PH**_		***N***	***H***_**O**_**±S.E.**	***F***_**IS**_	***N***_**A**_	***N***_**PA**_	***A***_**R**_	**Sib**
L. Winnipeg												
A. Cedar L.	25	0.77±0.01	6	2		25	0.63±0.04	0.025	36	0	3.35	0.12
B. L. Winnipeg	25	0.53±0.01	3	1		25	0.67±0.02	0.003	38	0	3.44	0.08
C. L. of the Woods	25	0.33±0.02	2	0		30	0.64±0.03	0.099	39	0	3.68	0.00
D. McKim L.	25	0.61±0.02	4	0		25	0.57±0.04	-0.021	34	0	3.00	0.28
Upper Mississippi R.												
E. Mille Lacs	25	0.16±0.02	3	0		39	0.62±0.02	0.010	38	2	3.22	0.15
L. Superior												
F. St. Louis R.	25	0.50±0.01	2	0		28	0.68±0.02	0.116	56	0	4.20	0.14
G. L. Nipigon	25	0.23±0.02	3	1		30	0.74±0.03	-0.047	43	0	3.76	0.40
H. Portage L.	25	0.64±0.01	3	0		56	0.73±0.02	0.001	52	0	3.97	0.07
L. Michigan												
I. Muskegon R.	25	0.23±0.02	3	0		50	0.73±0.01	0.057	57	0	4.20	0.04
L. Huron												
J. Thunder Bay	25	0.55±0.02	3	0		40	0.70±0.01	0.015	55	2	3.88	0.15
K. Flint R.	25	0.58±0.02	5	0		44	0.77±0.01	-0.023	55	0	4.15	0.09
L. Moon/Musquash R.	25	0.15±0.02	2	0		35	0.71±0.03	0.024	49	0	3.87	0.14
L. St. Clair												
M. Thames R.	25	0.72±0.01	4	0		39	0.75±0.02	0.012	63	0	4.19	0.05
N. Detroit R.	95	0.74±0.00	6	1		123	0.71±0.01	0.022	72	2	4.06	0.07
N1. Belle Isle	25	0.78±0.01	5	0		40	0.72±0.02	0.010	62	0	4.06	0.00
N2. Fighting Is.	45	0.73±0.01	6	0		48	0.68±0.01	0.044	57	0	3.92	0.08
N3. Grosse Ile	25	0.77±0.01	4	0		35	0.74±0.02	0.001	60	0	4.20	0.11
Contemporary L. Erie												
O. Western L. Erie	100	0.76±0.00	9	3		211	0.70±0.01	0.035	78	0	4.02	0.07
O1. Huron R.	25	0.78±0.01	5	1		40	0.73±0.02	0.021	64	0	4.18	0.00
O2. Hen Is.	25	0.78±0.01	5	0		65	0.67±0.01	0.039	63	0	3.84	0.09
O3. Maumee R.	25	0.72±0.01	4	0		76	0.69±0.01	0.042	65	0	4.01	0.11
O4. Sandusky R.	25	0.76±0.01	7	2		30	0.75±0.02	0.006	54	0	4.15	0.00
P. Eastern L. Erie	50	0.82±0.00	11	4		137	0.74±0.01	0.034	74	1	4.30	0.09
P1. Van Buren Bay	25	0.76±0.01	5	1		87	0.76±0.01	0.021	64	1	4.33	0.13
P2. Cattaraugus Ck.	25	0.88±0.01	9	3		50	0.71±0.02	0.053	66	0	4.18	0.04
Historic L. Erie												
Q. Historic yellow walleye	20	0.10±0.02	2	1		31	0.40±0.04	0.258	44	6	2.83	0.23
R. “Blue pike”	20	0.00±0.00	1	0		25	0.54±0.07	-0.191	20	0	2.06	0.76
L. Ontario												
S. Pigeon L.	25	0.47±0.02	4	0		29	0.73±0.02	-0.017	55	0	4.01	0.00
T. Bay of Quinte	25	0.62±0.02	5	1		50	0.69±0.02	0.046	65	0	4.00	0.08
U. Oneida L.	25	0.66±0.01	6	3		25	0.66±0.03	0.103	48	0	3.98	0.08
Lac Mistassini												
V. Lac Mistassini	25	0.48±0.01	2	0		40	0.52±0.03	0.137	45	2	3.09	0.05
Ohio R.												
W. Ohio R.	11	0.85±0.02	5	0		4	0.61±0.07	0.306	33	0	4.71	0.00
X. New R.	25	0.45±0.05	3	0		35	0.68±0.01	0.121	60	1	4.29	0.06
Tombigbee R.												
Y. North R.	5	0.40±0.11	2	1		5	0.56±0.11	0.197	27	3	3.53	0.00
Mean contemporary walleye (23 sites; A–P, S–Y)	31	0.53±0.02	4	1		49	0.68±0.03	0.055	39	1	3.86	0.08

Values include number of samples (*N*), haplotypic diversity (*H*_D_) and heterozygosity (*H*_O_) ± standard error (S.E.), number of haplotypes (*N*_H_) and alleles (*N*_A_), number of private haplotypes (*N*_PH_) and alleles (*N*_PA_), inbreeding coefficient (*F*_IS_), allelic richness (*A*_R_), and proportion of full siblings (Sib) determined from Colony v2.0.5.0 [55] analyses**.** Microsatellite results from these seven loci are statistically equivalent to those obtained from nine loci [30]. Latitude and longitude for each location are provided in Additional file 10.

Haplotype diversity (*H*_D_) varies greatly among populations (Table 1), with the Moon/Musquash Rivers (L) and Mille Lacs (E) being lowest and Ohio/New Rivers (W–X), eastern Lake Erie (P), Cedar Lake (A), and western Lake Erie (O) highest. Historic walleye and “blue pike” samples had much lower diversity than contemporary walleye from Lakes Erie and Ontario, supported by analysis of variance (ANOVA; *F* = 47.24, df = 2, *p* = 0.005).

### Genetic diversity patterns from nuclear DNA loci

All nine μsat loci conform to Hardy Weinberg Equilibrium (HWE) expectations and are unlinked. Locus *Svi*17 has a higher *F*_ST_ value, suggesting some possible positive selection, whereas *Svi*L7 has the lowest, suggesting slight balancing selection based on results from Lositan [56] (Additional file 3). The remaining seven loci conform to neutrality, having intermediate *F*_ST_ values (mean = 0.090). Locus *Svi*18 has the fewest alleles and *Svi*6 the most. Seven loci are included here for historic walleye and “blue pike”, since *Svi*17 and L7 failed to amplify. Results from the seven versus the nine loci are identical among the 23 contemporary spawning groups, therefore, the former are presented here; the dataset based on nine loci was detailed by Stepien et al. [30,40].

Most walleye populations (87%, 152/175 comparisons) are free of null alleles. Micro-Checker v2.2.3 [57] detects some slight homozygote excess at *Svi*33 (for populations C, L, V), *Svi*4 (C, P), *Svi*18 (O, Q, R, V), *Svi*L6 (O, Q, T), *Svi*6 (F, Q, U, X), and *Svi*7 (L, M, Q). Since those loci have no such excess in other spawning groups, all populations are in HWE, and are free of heterozygote deficiency, scoring error, and stuttering [57], all loci are analyzed here. The historic walleye sample (Q) contains some possible null alleles (57%, *Svi*18, L6, 6, and 7), which appear more prevalent at loci having longer allelic lengths (Additional file 4). However, these allelic frequencies are similar among all samples: 0.00–0.20 for contemporary walleye spawning groups, 0.02–0.22 for historic walleye (site Q) and 0.00–0.27 for historic “blue pike” (R), negating their possible bias.

FreeNA analysis [58] results moreover discern no effect from possible null alleles on *F*_ST_ values. With FreeNA corrections, pairwise comparisons between the historic (Q–R) and contemporary samples (O–P, S–T) only slightly change (from 0.141 to 0.120), retaining a similarly large magnitude of difference between the sampling intervals. *F*_ST_ values between the contemporary populations change only at the thousandth decimal point after correction (e.g., 0.058 to 0.054). Thus, there was no need to adjust the allele frequencies against possible null alleles, as there was no apparent effect.

We recover 155 alleles among seven nuclear μsat loci, across 23 contemporary walleye spawning groups, historic walleye, and “blue pike” (Table 1, Additional file 3). Many alleles are shared, widely distributed, and have high frequency in contemporary and historic samples (Additional file 4). Great Lakes’ populations possess more alleles than the others, with Lake Erie housing the most, similar to control region data. Allelic richness is highest in the Ohio (W/X) and North Rivers (Y), moderate in Great Lakes’ populations, and lowest in the northern sites (A–E, V). ANOVA indicates that allelic richness significantly varies among populations (*F* = 2.50, df = 22, *p* < 0.001); Tukey’s *post hoc* tests, however, reveal no overall pattern.

Historic walleye (Q) and “blue pike” (R) possess fewer alleles and lower allelic richness (ANOVA *F* = 11.129, df = 5, *p* < 0.001) than characterize contemporary walleye from Lakes Erie (sites O–P) and Ontario (S–T; Table 1, Additional file 4). Allele lengths show some tendency to be smaller in historic samples and longer in the contemporary samples (Additional file 4). Historic walleye also display some inbreeding depression, whereas “blue pike” suggest some outbreeding (Table 1), but both were in HWE. Contemporary populations comparatively have moderate values. Colony v2.0.5.0 [55] analyses indicate that full siblings constitute 8% of contemporary walleye populations, being highest in McKim Lake (D) and Lake Nipigon (G). Higher values also characterize our historic walleye and “blue pike” samples (R).

Modest proportions of private μsat alleles typify the 23 contemporary samples, with the North River (Y) having the highest (Table 1). Historic walleye samples contained the most private alleles. However, all historic “blue pike” samples possessed common walleye alleles, with no unique ones, supporting lack of distinction.

Genetic diversity values from the μsat loci (*H*_O_) generally are somewhat higher than in the control region (Table 1). Diversities of historic walleye and “blue pike” were relatively low compared to contemporary samples. ANOVA indicates some differences in diversity among contemporary spawning groups (*F* = 1.79, df = 22, *p* = 0.02), and between contemporary versus historic samples (*F* = 3.83, df = 5, *p* = 0.007), however, Tukey’s tests describe no overall pattern. Thus, genetic diversity is similar among all samples.

### Spatial genetic structure of walleye populations

Relationships among spawning groups show a pattern of genetic isolation with geographic distance (Additional file 5). Most are genetically distinctive (Table 2, Additional file 6) for both the control region and μsat data sets, indicating high genetic structure. The most pronounced divergences distinguish three geographic regions: northern (A–E), Great Lakes (F–U), and southeastern (X–Y). Notably, the Ohio/New (W–X) and North River (Y) populations differ by the greatest *F*_ST_ values. Geneclass2 [59] assignment tests denote high self-assignment among spawning groups and to the three geographic regions, similar to pairwise comparison results (Additional file 7). Hierarchical analysis of molecular variance (AMOVA) [60] also supports division into three geographic regions: northern (A–E, V), Great Lakes (F–U), and southeast (W–Y), which explains the most overall variation, has highest mean *F*_ST_ values, and best characterizes the data. A second well-supported scenario distinguishes walleye from each of the 11 drainage systems, with high mean *F*_ST_ values, but less support. The scenario for potential partitioning between river and lake population groups is unsupported.

**Table 2:** **Genetic *F***_**ST **_**divergences between pairs of walleye population samples, including contemporary spawning groups (labeled A–P, S–Y), historic Lake Erie walleye (Q) and “blue pike” (R), from mtDNA control region sequences (below diagonal) and nuclear μsat data (above diagonal)**

**Location**	**A.**	**B.**	**C.**	**D.**	**E.**	**F.**	**G.**	**H.**	**I.**	**J.**	**K.**	**L.**	**M.**	**N.**	**O.**	**P.**	**Q.**	**R.**	**S.**	**T.**	**U.**	**V.**	**W.**	**X.**	**Y.**
A. Cedar L.	---	**0.059**	**0.054**	**0.136**	**0.152**	**0.084**	**0.080**	**0.112**	**0.102**	**0.095**	**0.087**	**0.144**	**0.111**	**0.129**	**0.123**	**0.115**	**0.252**	**0.318**	**0.123**	**0.124**	**0.122**	**0.188**	*0.076*	**0.109**	**0.249**
B. L. Winnipeg	*0.164*	---	**0.028**	**0.150**	**0.144**	**0.080**	**0.040**	**0.111**	**0.119**	**0.144**	**0.122**	**0.155**	**0.127**	**0.141**	**0.131**	**0.113**	**0.240**	**0.281**	**0.155**	**0.143**	**0.136**	**0.189**	0.039	**0.128**	**0.252**
C. L. of the Woods	**0.248**	*0.271*	---	**0.147**	**0.090**	**0.064**	*0.031*	**0.081**	**0.093**	**0.108**	**0.093**	**0.124**	**0.100**	**0.113**	**0.109**	**0.091**	**0.222**	**0.267**	**0.128**	**0.121**	**0.125**	**0.162**	0.034	**0.104**	**0.223**
D. McKim L.	*0.071*	*0.174*	0.037	---	**0.222**	**0.168**	**0.216**	**0.181**	**0.212**	**0.238**	**0.216**	**0.234**	**0.221**	**0.230**	**0.222**	**0.195**	**0.384**	**0.440**	**0.253**	**0.244**	**0.203**	**0.239**	**0.213**	**0.211**	**0.334**
E. Mille Lacs	**0.360**	**0.487**	0.072	*0.136*	---	**0.115**	**0.069**	**0.149**	**0.154**	**0.155**	**0.162**	**0.177**	**0.115**	**0.115**	**0.111**	**0.097**	**0.274**	**0.330**	**0.184**	**0.139**	**0.150**	**0.183**	**0.155**	**0.117**	**0.288**
F. St. Louis R.	*0.171*	0.000	*0.256*	*0.167*	**0.480**	---	**0.053**	**0.035**	**0.044**	**0.055**	**0.046**	**0.061**	**0.034**	**0.045**	**0.040**	**0.029**	**0.179**	**0.211**	**0.087**	**0.067**	**0.050**	**0.138**	0.003	**0.044**	**0.164**
G. L. Nipigon	**0.385**	*0.173*	**0.646**	**0.501**	**0.801**	*0.203*	---	**0.086**	**0.088**	**0.094**	**0.088**	**0.119**	**0.073**	**0.082**	**0.077**	**0.069**	**0.190**	**0.242**	**0.110**	**0.093**	**0.108**	**0.168**	0.031	**0.085**	**0.215**
H. Portage L.	**0.173**	**0.280**	*0.167*	*0.094*	**0.236**	**0.280**	**0.557**	---	**0.044**	**0.066**	**0.047**	**0.036**	**0.043**	**0.055**	**0.054**	**0.044**	**0.169**	**0.200**	**0.090**	**0.074**	**0.044**	**0.130**	0.014	**0.051**	**0.212**
I. Muskegon R.	**0.334**	**0.446**	0.054	*0.117*	0.000	**0.439**	**0.765**	*0.187*	---	**0.028**	*0.007*	**0.052**	**0.031**	**0.046**	**0.042**	**0.041**	**0.095**	**0.135**	**0.047**	**0.041**	**0.042**	**0.167**	0.008	**0.031**	**0.156**
J. Thunder Bay	**0.196**	**0.301**	*0.098*	*0.064*	*0.145*	**0.297**	**0.603**	0.000	*0.098*	---	*0.011*	**0.073**	**0.027**	**0.036**	**0.035**	**0.040**	**0.119**	**0.184**	**0.036**	**0.031**	**0.066**	**0.196**	0.035	**0.042**	**0.188**
K. Flint R.	**0.177**	**0.270**	0.108	*0.064*	*0.175*	**0.266**	**0.571**	0.002	*0.119*	0.000	---	**0.046**	**0.020**	**0.039**	**0.037**	**0.037**	**0.122**	**0.171**	**0.030**	**0.033**	**0.053**	**0.177**	0.010	**0.034**	**0.180**
L. Moon/Musquash R.	**0.376**	**0.489**	0.078	*0.152*	0.000	**0.483**	**0.803**	*0.210*	0.000	*0.114*	*0.138*	---	**0.045**	**0.062**	**0.059**	**0.052**	**0.209**	**0.249**	**0.083**	**0.064**	**0.063**	**0.160**	*0.041*	**0.061**	**0.220**
M. Thames R.	**0.165**	**0.280**	**0.258**	*0.152*	**0.339**	**0.285**	**0.518**	0.000	**0.283**	0.046	0.030	**0.315**	---	0.001	0.000	0.002	**0.150**	**0.199**	**0.040**	**0.013**	**0.035**	**0.157**	0.011	*0.008*	**0.184**
N. Detroit R.	**0.158**	**0.253**	**0.213**	**0.138**	**0.259**	**0.255**	**0.437**	0.011	**0.221**	*0.053*	*0.041*	**0.243**	0.000	---	0.001	**0.007**	**0.142**	**0.191**	**0.045**	**0.020**	**0.058**	**0.166**	*0.035*	**0.021**	**0.198**
O. Western L. Erie	**0.148**	**0.242**	**0.200**	**0.126**	**0.244**	**0.243**	**0.426**	0.007	**0.208**	*0.048*	*0.049*	**0.233**	0.000	0.000	---	**0.006**	**0.136**	**0.182**	**0.048**	**0.017**	**0.048**	**0.160**	0.024	**0.018**	**0.198**
P. Eastern L. Erie	**0.137**	**0.243**	**0.253**	**0.154**	**0.314**	**0.249**	**0.422**	0.039	**0.275**	*0.095*	*0.102*	**0.308**	0.008	0.010	0.000	---	**0.144**	**0.185**	**0.050**	**0.026**	**0.040**	**0.136**	0.019	**0.011**	**0.173**
Q. Historic walleye	**0.541**	**0.668**	**0.773**	**0.627**	**0.869**	**0.683**	**0.830**	**0.499**	**0.824**	**0.581**	**0.516**	**0.860**	**0.352**	**0.304**	**0.336**	**0.364**	---	**0.050**	**0.140**	**0.132**	**0.155**	**0.343**	*0.139*	**0.149**	**0.276**
R. “Blue pike”	**0.585**	**0.712**	**0.817**	**0.672**	**0.913**	**0.728**	**0.875**	**0.550**	**0.870**	**0.632**	**0.567**	**0.908**	**0.405**	**0.334**	**0.366**	**0.399**	0.000	---	**0.222**	**0.196**	**0.196**	**0.384**	**0.203**	**0.190**	**0.367**
S. Pigeon L.	**0.351**	**0.471**	**0.541**	**0.402**	**0.635**	**0.484**	**0.651**	*0.247*	**0.583**	**0.322**	*0.253*	**0.610**	*0.119*	**0.136**	**0.171**	**0.194**	*0.092*	*0.145*	---	*0.010*	**0.082**	**0.225**	0.031	**0.055**	**0.196**
T. Bay of Quinte	**0.272**	**0.392**	**0.455**	**0.319**	**0.547**	**0.403**	**0.576**	*0.155*	**0.495**	*0.233*	*0.182*	**0.522**	0.046	*0.073*	*0.102*	*0.112*	*0.151*	*0.205*	0.000	---	**0.056**	**0.198**	**0.030**	0.032	**0.198**
U. Oneida L.	**0.269**	**0.405**	**0.503**	**0.367**	**0.584**	**0.420**	**0.557**	**0.289**	**0.532**	**0.350**	**0.295**	**0.581**	*0.169*	**0.150**	**0.161**	**0.138**	**0.400**	**0.454**	**0.240**	*0.186*	---	**0.126**	0.020	*0.018*	**0.188**
V. L. Mistassini	*0.163*	0.088	0.022	0.027	*0.226*	0.072	**0.463**	*0.161*	*0.191*	*0.131*	*0.119*	*0.230*	**0.218**	**0.191**	**0.179**	**0.211**	**0.694**	**0.739**	**0.473**	**0.389**	**0.430**	---	**0.177**	**0.136**	**0.324**
W. Ohio R.	*0.116*	*0.259*	*0.293*	*0.142*	**0.413**	*0.269*	**0.531**	0.068	*0.346*	*0.118*	*0.116*	**0.416**	0.041	0.034	0.020	0.004	**0.557**	**0.629**	**0.287**	*0.180*	*0.151*	*0.222*	---	0.010	*0.122*
X. New R.	**0.382**	**0.508**	**0.607**	**0.470**	**0.693**	**0.523**	**0.660**	**0.443**	**0.653**	**0.492**	**0.467**	**0.695**	**0.384**	**0.338**	**0.331**	**0.310**	**0.685**	**0.732**	**0.512**	**0.438**	**0.364**	**0.533**	*0.169*	---	**0.171**
Y. North R.	**0.342**	**0.506**	**0.651**	**0.454**	**0.793**	**0.527**	**0.736**	**0.432**	**0.732**	**0.495**	**0.468**	**0.798**	**0.369**	**0.338**	**0.327**	**0.288**	**0.836**	**0.923**	**0.551**	**0.447**	*0.361*	**0.541**	*0.299*	**0.543**	---

Results are congruent to those from exact tests of differentiation (Additional file 6). Values using these seven nuclear μsat loci data are almost identical to those based on nine loci, differing only at the thousandth decimal place (see Stepien et al. [30] for the nine locus dataset). **Bold** = significant before and following sequential Bonferroni corrections, *italics* = significant at α = 0.05, and normal text = not significant.

**Figure 3:**
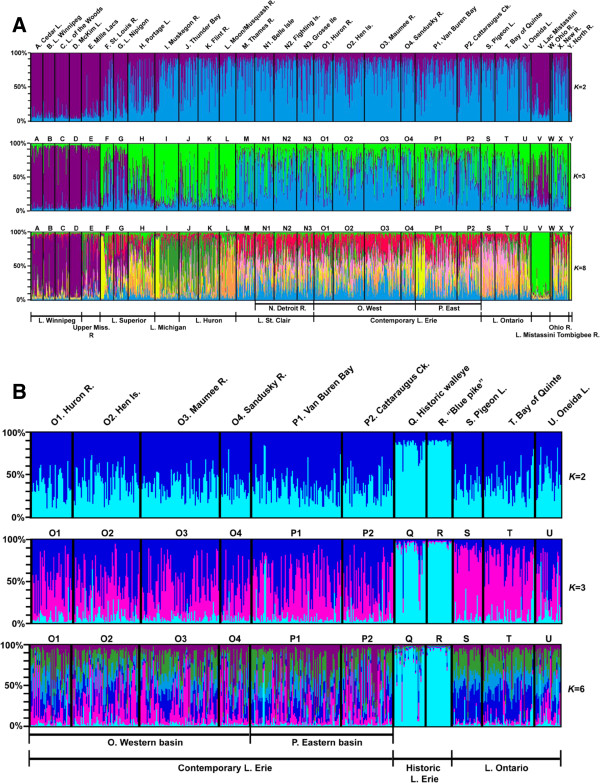
**Bayesian Structure analysis [**[61]**] for A contemporary walleye (*K*** **= 2, 3, 8) and B contemporary Lakes Erie and Ontario and historic Lake Erie walleye (with “blue pike”; *K*** **= 2, 3, 6) population groups, based on seven μsat loci.** Individuals are represented as thin vertical lines, partitioned into *K* colored segments.

Barrier v2.2 [26] and Structure v2.3.4 [61] analyses likewise identify significant genetic structuring across the range (Figures 1 and 3, Additional file 8). The first mtDNA barrier separates the southern (W–Y) from northern populations (A–P, S–V), the second isolates the northernmost sites (A–G, V), and the third distinguishes Lake Nipigon (G). In contrast, barriers for μsat loci denote finer-scale discontinuities, with the first separating Lake Nipigon (G, barrier I; 49% bootstrap support, 7/7 loci), then the North River (Y, II; 57%, 7/7), followed by Lac Mistassini (V, III; 43%, 6/7). Remaining barriers for both data sets depict fine-scale divergences, with most among northern spawning groups (Figure 1). Structure analyses indicate high assignment of the northernmost populations (A–G, V, purple color) from those to the south (I–U, W–Y, blue; Figure 3A). Populations in the upper (F–L; orange) and lower (M–U; light blue) Great Lakes assign separately, with St. Louis River (F) and Lake Nipigon (G) walleye clustering more closely to those in the northwest. Finer-scale demarcations by drainage and some spawning groups also are recovered, similar to results from the analyses using pairwise comparisons, Geneclass, and AMOVA.

The combined population genetic distance tree (Additional file 9) also shows separation of the southern samples, with the North River (Y) walleye as basal, matching its high pairwise divergence (Table 2). The tree clusters northern walleye and those from Lake Superior together (sites A–G, V; 62%), similar to Barrier results. Lakes St. Clair (M–N) and Erie populations (O–P) display close genetic relationship, analogous with pairwise comparisons.

### Historic vs. contemporary populations of Lake Erie walleye

Historic walleye and “blue pike” samples are indistinguishable in control region sequences, and vary from each other in μsat allele frequencies within the realm among contemporary walleye populations, on the low end among the 23 spawning groups (Table 2, Additional file 6). In contrast, greater values distinguish walleye populations spawning in the New (X) and North Rivers (Y). Thus, there is no genetic evidence that the “blue pike” comprised a distinct taxon from walleye.

Both sets of historic samples differed in haplotypic and allelic frequencies from contemporary Lakes Erie and Ontario walleye in the *F*_ST_ and *χ*^2^ analyses. The historic samples significantly varied in genetic composition from all but one contemporary spawning group (Table 2, Additional file 6). The control region frequencies of the Pigeon Lake spawning group (S) alone resemble the historic samples from Lake Erie due to shared high representation of haplotype 3, but differ in the μsat data. This likely is an artifact.

No genetic distinction occurs between turquoise-colored mucus versus yellow walleye from McKim Lake (D, control region: *F*_ST_ = 0.000, *χ*^2^ = 0.00, μsat: *F*_ST_ = 0.000, *χ*^2^ = 14.05). Together, McKim Lake represents a distinct spawning group, differing from other walleye populations (Table 2, Additional file 6). The historic walleye and “blue pike” samples from Lake Erie were very significantly different from the McKim Lake samples. Thus, both turquoise mucus walleye and “blue pike” are walleye.

AMOVA analyses show no significant difference between the historic (walleye and “blue pike” considered together as a single group) versus the contemporary samples for control region sequences. The historic and contemporary samples differ with μsat data (Table 3) by ~2–5x greater than the mean *F*_ST_ values among contemporary walleye spawning groups. Results thus indicate that μsat allelic frequencies have changed from the past to the present.

**Table 3:** Relative distribution of genetic variation among contemporary and historic walleye samples using AMOVA, calculated from mtDNA control region sequence and nuclear DNA μsat data

	**Control region**			**Microsatellite loci**		
**Source of variation**	**% variation**	**Φ value**	**Mean *F***_**ST**_	**% variation**	**Φ value**	**Mean *F***_**ST**_
1. Contemporary (O–P,S–T) vs. historic samples (Q–R)	26.01	0.260 NS	0.260	6.99	0.070 NS	0.167
Among sampling sites within groups	0.00	0.000 NS	0.176	0.87	0.009**	0.030
Within sampling sites	73.98	0.252**	---	92.14	0.079**	---
2. Among northern (A–E,V), Great Lakes (F–U), and southeast (W–Y) regions	12.06	0.121**	0.318	5.07	0.051**	0.129
Among sampling sites within groups	17.64	0.201**	0.238	4.97	0.052**	0.063
Within sampling sites	70.30	0.297**	---	89.96	0.100**	---
3. Among the contemporary 11 drainages (A–P,S–Y)	14.38	0.144**	0.286	4.26	0.043**	0.132
Among sampling sites within drainages	11.07	0.129**	0.158	3.13	0.033**	0.058
Within the sampling sites	74.05	0.254**	---	92.61	0.074**	---
4. Between lake (A–E,G–H,J,O2,P1,S–V) and river (F,I,K–O1,O3–4,P2,W–Y) spawners	0.00	0.000 NS	0.306	0.58	0.006**	0.107
Among sampling sites within groups	24.92	0.247**	0.298	6.59	0.066**	0.100
Within sampling sites	75.08	0.241**	---	92.83	0.071**	---

NS = not significant, ** = significant.

The genetic distance tree of the combined gene data (Additional file 9) and Structure analyses (Figure 3B, Additional file 8) both separate the historic samples into a distinct group. Geneclass assignment tests likewise depict their genetic difference, with 89% of the historic samples self-assigning. Just 10% mis-assign to the contemporary eastern Lake Erie walleye (group P), 6% to historic “blue pike” (R) and 6% to contemporary western Lake Erie walleye (O). Historic “blue pike” individuals self-assigned 48% of the time (*N* = 12), with 48% mis-assigning to historic walleye. A single “blue pike” individual assigned to contemporary eastern Lake Erie walleye (4%).

### Morphological traits of historic samples

Morphological variations occur between walleye and “blue pike” in six characters; “blue pike” individuals tend to have the smallest head and interorbital widths, largest eye diameter, highest numbers of pelvic fin rays, a larger orbit: interorbital ratio, and fewer second dorsal fin rays (Table 4). However, the ranges of all characters extensively overlap and do not reliably distinguish a “blue pike” from a walleye specimen.

**Table 4:** **Morphological data from historic walleye and** “**blue pike” samples, with mean, standard deviation (SD), and range of values**

	**Historic walleye (*N*** **= 51)**		**“Blue pike” (*N*** **= 52)**	
**Measurement**	**Mean ± SD**	**Range**	**Mean ± SD**	**Range**
Standard length (mm)	264.3 ± 49.9	187 - 385	248.8 ± 37.7	202 - 362
% Standard length				
Body depth	20.1 ± 1.9	15.8 - 24.2	20.4 ± 2.1	16.3 - 24.9
Head length	30.7 ± 0.8	28.9 - 32.2	30.6 ± 1.8	27.9 - 40.7
% Head length				
Cheek length	48.3 ± 1.8	44.3 - 52.5	48.4 ± 1.6	46.3 - 55.7
Upper jaw length	38.6 ± 3.1	33.6 - 43.9	37.9 ± 3.7	31.0 - 44.9
Lower jaw length	38.8 ± 4.1	30.1 - 43.7	38.2 ± 4.3	31.6 - 44.1
Head width	41.6 ± 4.5	34.3 - 55.0	38.6 ± 2.5	32.1 - 44.0**
Interorbital width	14.9 ± 1.1	12.8 - 17.3	14.1 ± 1.2	11.2 - 17.2**
Orbit diameter	20.7 ± 1.8	17.4 - 26.4	21.9 ± 2.1	16.6 - 25.9**
Orbit: interorbital	1.4 ± 0.2	1.1 - 2.0	1.6 ± 0.2	1.0 - 2.2**
Upper: lower jaw	1.0 ± 0.2	0.8 - 1.3	1.0 ± 0.2	0.7 - 1.3
Meristic	Mode	Range	Mode	Range
First dorsal fin rays	14	11 - 16	12	11 - 15
Second dorsal fin rays	21	19 - 22	20	13 - 22**
Pectoral fin rays	15	11 - 17	14	10 - 16
Pelvic fin rays	6	5 - 8	7	5 - 8**
Anal fin rays	14	11 - 16	14	11–16

Body and head measurements are given as percentage of standard or head lengths, respectively. Ratios are between the original measurement values.

Additional file 11 lists individuals examined. ** = statistically significant comparisons between walleye and “blue pike” using Student’s *t* and Mann Whitney *U* tests. Overall, walleye and “blue pike”differed according to the nonparametric MANOVA (morphometric: *F* = 4.34, degrees of freedom (df) = 1, *p* < 0.001; meristic: *F* = 4.13, df = 1, *p* = 0.001).

**Figure 4:**
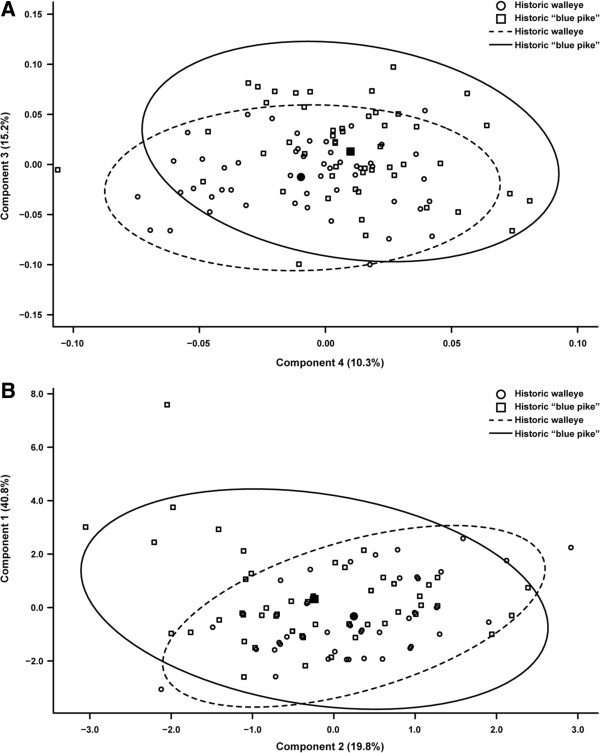
**Principal components analysis plots for historic walleye and “blue pike”, based on A morphometric and B meristic characters.** Solid symbols = mean component scores. Circles = 95% confidence intervals for each group.

The first four morphometric principal components (PC) explain 86% of the overall variation and the first three meristic PCs explain 79% between historic walleye and “blue pike”. Multivariate analysis of variance (MANOVA) shows significant difference in morphometric (Wilks’ Λ = 0.768, *F* = 7.244, df = 1, *p* < 0.001) and meristic principal components analysis (PCA) (Wilks’ Λ = 0.876, *F* = 4.522, df = 1, *p* = 0.005; Figure 4). Morphometric PC3 and 4 distinguish walleye and “blue pike” (ANOVA, PC3: *F* = 10.443, df = 1, *p* = 0.002, PC4: *F* = 9.004, df = 1, *p* = 0.003) and are correlated with body depth (*r* = 0.850) and head width (*r* = -0.546). Meristic PC1 and 2 (Figure 4) also differentiate them (ANOVA, PC1: *F* = 4.323, df = 1, *p* = 0.040, PC2: *F* = 5.0832, df = 1, *p* = 0.026) in number of second (*r* = -0.827) and anal fin rays (*r* = -0.578).

Quadratic discriminant function analysis (DFA) correctly assigns 72% of samples using the morphometric dataset and just 64% with meristic characters. Historic walleye (76% morphometric, 71% meristic data) and “blue pike” (68%, 57%) more often self-assign, but have high mis-assignment to each other, similar to the Geneclass results. Thus our findings do not support designation of “blue pike” as a subspecies or species, since it lacks reliable morphological characters and has no genetic differentiation, with no unique haplotypes or alleles. Its level of frequency difference in μsat allelic composition appears typical of a walleye population.

## Discussion

### Broadscale genetic divergence patterns

Walleye populations from the unglaciated southern portion of the range (W–Y) are the most divergent, possessing unique alleles and haplotypes. The earliest and most divergent haplotype (19) occurs in the Ohio and New Rivers (W–X), and is very genetically distinct ([30,62] this study). Other fishes, including brook trout *Salvelinus fontinalis* (Mitchill 1814) [63], smallmouth bass *Micropterus dolomieu* Lacepède 1802 [64], greenside darter *Etheostoma blennioides* Rafinesque 1819 [65], rainbow darter *Etheostoma caeruleum* Storer 1845 [66], and yellow perch [67], likewise demonstrate pronounced population divergence of Atlantic coastal groups, indicating long-term isolation.

The North River (Y), which drains south into the Gulf of Mexico, is dominated by an endemic walleye haplotype (20). The population is small, very isolated, and relatively low in diversity, as previously documented [25,30]. Boschung and Mayden [68] noted that this indigenous North River population is at risk of colonization from northerly walleye due to connections through the Tombigbee**–**Tennessee River waterway, which may threaten localized adaptations. Likewise, yellow perch from the Gulf Coastal drainage possessed high endemism, unique haplotypes, and extensive divergence [67]. Southerly populations of walleye and yellow perch thus represent unique genetic sources that may prove valuable for conservation.

Walleye populations exhibit moderate divergence levels across the range, as described by previous studies [30,41,69]. In contrast, yellow perch (control region mean *F*_ST_ = 0.469, μsat = 0.236) [67] and smallmouth bass (cytochrome *b* mean *F*_ST_ = 0.412, μsat = 0.232) [64,70] possess much higher divergence among spawning groups. This may be due to their more limited migration [71,72]. Walleye have been documented to disperse 50–300 km [73], followed by yellow perch to 48 km, with occasional individuals travelling 200 km [71], and smallmouth bass only to ~10 km [72].

The European perch *P. fluviatilis* Linnaeus 1758 discriminates kin from non-kin via olfactory cues and schools in family groups, which may reproduce together [74,75]. This life history pattern remains to be tested for yellow perch, walleye, or smallmouth bass. Kinship tests by our laboratory reveal high proportions of full siblings in some spawning groups of yellow perch (mean = 0.20 ranging to 75%) [76] and smallmouth bass (0.15, to 67%) [70], which are greater than those identified here for walleye. Limited lifetime migration, and apparent close association among kin for yellow perch and smallmouth bass, may lead to their higher divergences among proximate populations. Walleye populations display intermediate divergence and higher diversity, attributable to more outbreeding. Thus, biogeographic patterns of these widely distributed freshwater fishes largely result from a combination of extrinsic (i.e., changes in climate and drainage patterns) and intrinsic factors (dispersal capabilities, degree of natal homing, population size, and inbreeding), which are regulated by behavior of the species.

### Contemporary genetic diversity patterns

Our data indicate relatively high genetic diversity for most walleye spawning groups; these values mirror those reported across their range using nine μsat loci (*H*_O_ *=* 0.68) [30]. Difference between the mtDNA and nuclear data is attributable to the former having a slower evolutionary rate [51,77], ¼ effective the population size, and being more influenced by population bottlenecks [50].

Walleye reproducing in the lower Great Lakes (Lakes St. Clair and Erie) have the highest diversity values, reflecting admixture from glacial refugia and larger population sizes, similar to results from other studies [30,40]. Some small upper Great Lakes populations display the lowest mtDNA diversities, but average nuclear DNA diversities. Notably, the population from the Moon/Musquash River (site L) has the least control region diversity but higher μsat value. Gatt et al. [78] also recovered low mtDNA diversity at this location, attributing this to stocking and overexploitation. Since this pattern is restricted to mtDNA alone in our study, it likely instead reflects past bottlenecking and small population size.

Yellow perch populations possess much lower mtDNA control region diversity levels across their range (mean *H*_D_ *=* 0.31) than nuclear DNA variability (*H*_O_ *=* 0.53, 15 μsat loci) [67]. This also is true for the related European perch [79] and Eurasian ruffe *Gymnocephalus cernua* (Linnaeus 1758) [80]. This difference among percid species may reflect their respective evolutionary history and behavior. Notably, strong association of European perch in kin groups [74,75] and high proportions of yellow perch full siblings in spawning groups [76] may lead to lower diversity from inbreeding. Smallmouth bass spawning groups also have lower genetic diversity than walleye in mtDNA sequences (mean *H*_D_ *=* 0.50) [70] and eight μsat loci (*H*_O_ *=* 0.46) [64]. This may reflect higher association of kin groups and limited lifetime migration [70]. Thus, population genetic diversity and divergence of smallmouth bass [64,70] and yellow perch [76] differ from walleye due to their respective reproductive behavior and life history characters.

Small isolated southern populations of walleye have lower genetic diversity values than those in once glaciated regions. The former contain the oldest and most unique genetic variants, reflecting long-term isolation, smaller population sizes, and likely genetic drift and population bottlenecks [30]. Gulf Coastal yellow perch populations also possess the lowest diversity values, along with unique haplotypes and alleles [67], similar to walleye. These southern populations comprise a historic source of diversity and an important genetic resource for both species. Their adaptations to warmer habitats may provide a critical genetic reservoir in the face of climate warming.

### Genetic patterns shaped by anthropogenic factors in Lake Erie

Genetic diversity values for mtDNA and μsat loci are 94% and 37% lower for historic Lake Erie walleye and “blue pike” from 1923–49 compared with contemporary Lakes Erie and Ontario populations. Genetic diversity appears to have increased over the past 70+ years, which may reflect population recovery.

Europeans settled Lake Erie shores during the 1700–1800s, cutting down the forests and draining the marshlands, which disappeared by 1900 [44,81]. As the region developed and industrialized, untreated wastes were released into the Lake from saw mills, slaughterhouses, and steel factories. By 1830, Lake Erie walleye comprised an abundant and important commercial fishery. In 1874, construction of a major shipping channel drastically modified the Lakes Huron–Erie Corridor connection (Figure 1). Lake Erie steadily lost much of its fish habitat from 1900–1970s due to draining of wetlands, armoring of shorelines, channelization, dredging, and increased industrialization [82,83]. During the 1960s, high levels of phosphorus caused massive algal blooms, accompanied by oxygen depletion and marked fish die offs [82].

Many native Lake Erie fish populations experienced steady declines from 1900–1970s, including the lake trout, lake sturgeon *Acipenser fulvescens* Rafinesque 1817*,* and walleye [81,84]. Industrial outputs resulted in heavy metal contamination and declining fish health, manifested with neoplasms, tumors, and lesions on walleye and other species [85]. In 1970, walleye fisheries from Lakes Huron through Erie were closed due to high mercury levels. By 1978, fisheries managers declared Lake Erie walleye as being in crisis from overfishing and pollution [82].

Lower allelic numbers and diversity for historic walleye and “blue pike” from 1923–49 likely reflect these population declines. The rare haplotypes and alleles we identify in historic walleye may have disappeared. Most haplotypes and almost all alleles in historic Lake Erie samples are common and widespread today. The historic samples appeared to possess more haplotypes that trace to the Atlantic Refugium, whereas those from the Mississippian Refugium dominate today’s walleye populations [21,25,41]. This change merits further testing, but may be a response to climate warming.

Genetic diversity of other Great Lakes fishes similarly was lower in 1927–59 and higher in 1995–2005, attributed to population declines from environmental conditions and overexploitation [86,87]. Notably, lake whitefish *Coregonus clupeaformis* (Mitchill 1818) from Lakes Huron and Erie in 1927 had lower diversity at seven μsat loci (0.60) than in 1997–2005 (0.65) [87]. Lake trout from Lakes Superior, Michigan, and Huron were less variable in 1940–59 (0.47) than in 1995–99 (0.51) using five μsat loci [86]. Similarly, Atlantic cod *Gadus morhua* Linnaeus 1758 declined in diversity and number of alleles from 1954–80s, then increased from 1980–98 according to archived otolith samples and three μsat loci [88]. That study moreover documented that genetic composition changed, similar to the pattern here for Lake Erie walleye, hypothesizing recovered diversity via immigration from a nearby spawning group.

Contemporary Lake Erie walleye populations also may have been influenced by migration and recruitment. Walleye is described to natally home, with chemical cues presumably facilitating recognition of reproductive grounds [89,90]. Olson and Scidmore [89] discerned lower homing in areas with high habitat degradation. Some contemporary western Lake Erie spawning group samples appear genetically similar to those from Lake St. Clair, suggesting possible genetic exchange. Walleye movement between these lakes during non-spawning times is well known from tagging [91,92] and Lake Erie walleye may have migrated to spawn at a recently augmented reef in the Detroit River [53].

Lake Erie walleye appear to have recovered from declines in diversity and numbers (~10 million in 1978), after the fishery's closure from 1970–76 [93], implementation of the 1970 Canada Water Act, the 1972 U.S. Clean Water Act, and the 1972 Canada-U.S. Great Lakes Water Quality Agreement [82]. Increasing water temperature –especially in the shallow western basin– changed the Lake Erie fish community from cold water (e.g., lake trout) to warmer water species, favoring walleye and yellow perch. Declining numbers of colder water competitors presumably enhanced walleye abundance [84]. By 1988, Lake Erie had rebounded to ~80 million (8x 1978), decreasing to ~18 million walleye in 2013 [94]. Genetic analyses [38] revealed temporal consistency in genetic diversity from 1995–2008 for three primary Lake Erie walleye spawning groups (Maumee and Sandusky Rivers in the west, and Van Buren Bay in the east). Whether this continues remains to be discerned, as genetic variability may decline with population sizes.

Other walleye populations have shown a mixture of temporal stability and decline. Walleye spawning in Escanaba Lake, Wisconsin displayed consistent diversity levels from 1952–2002 (mean *H*_O_ = 0.76) using eight μsat loci [95] (six of those here). Garner et al. [96] likewise described consistent diversity levels for walleye in Lake Superior’s Black Bay from 1966–2010 (mean *H*_O_ = 0.62) employing nine μsat loci (six of those here). However, the Escanaba Lake and Black Bay populations were stocked, likely circumventing fluctuations. MtDNA restriction fragment length polymorphism diversity of walleye spawning in Lake Huron’s Georgian Bay declined over three decades (0.50 in the 1960s to 0.15 in the 1990s), attributed to exploitation and stocking [78]. Our study recovers similarly low mtDNA control region variability for walleye spawning in the Moon/Musquash Rivers of Georgian Bay today. However, this bottleneck effect is restricted to mtDNA, since we denote average levels of nuclear DNA variability.

Other alternatives may explain lower diversity levels in the historic samples. Our contemporary samples were adults collected from spring spawning runs at specific spawning sites. In contrast, historic samples were collected from July–November, when walleye intermix. Thus genetic diversity may have been lowered due to population admixture via a Wahlund effect [97]. For historically archived samples, such as ours, Nielsen and Hansen [98] recommended including positive and negative controls, having a separate laboratory space and separate chemicals, testing for null alleles with Micro-Checker, using samples with complete documentation of biological information, testing for HWE, and applying more than one statistical test to validate patterns. We followed all of these precautions to ensure reliability of data from our formalin fixed historic samples. Some studies have documented issues with historic samples having biased amplification of shorter length alleles [99,100]. We found slight suggestion of null alleles in historic samples, with shorter allele lengths being more prevalent. Lower template quality may have resulted from DNA shearing with formalin fixation [101], leading to partial repeat amplification if primer sites were unavailable for binding [102]. However, our Micro-Checker tests and other analyses demonstrate lack of statistical support for such problems. Our mtDNA sequences reveal the same pattern as the μsat analyses. Additional analysis of historic walleye and “blue pike” samples from intermediate decades may help to further interpret temporal population genetic patterns.

### Taxonomic status of historic “blue pike” and turquoise-mucus variants

We discern that the historic “blue pike” appears genetically indistinguishable from walleye populations. It has no unique genetic variation in our database, rendering *S. vitreus “glaucus”* invalid. It fails to meet the criteria of the Evolutionary Species Concept (ESC) [103] or the Phylogenetic Species Concept (PSC) [104]. The “blue pike” is not “an entity that kept its identity from others over time and space and that had its own independent evolutionary fate and historical tendencies” and possesses no bootstrap or posterior probability support, lacks reciprocal monophyly, diagnosable synapomorphies, and demarcation from the walleye as required by the ESC and PSC [103,104]. It also does not show interspecific variation 10x greater than the mean intraspecific variation of walleye [105]. In fact, the “blue pike” has no mtDNA sequence differentiation and its μsat variation is identical to that among typical walleye spawning groups and populations. In contrast, many walleye populations across North America possess much more pronounced genetic variation, particularly from the New (X) and North Rivers (Y). Walleye spawning in those southerly locations meet more of the criteria of being distinct taxa. However, we regard those in the New and North Rivers as divergent populations of walleye, and not as separate taxa, and believe that most ichthyologists and systematists would concur. Our findings thus indicate that the “blue pike” does not constitute a separate genetic taxon from walleye, and does not merit species or subspecies recognition.

Slight morphological variations between historic walleye and “blue pike” samples suggest some possible population-level differences. However, single individuals of the historic “blue pike” and walleye cannot be identified morphologically or genetically as either “blue pike” or walleye. Their coloration also is not a reliable identification character as it was/is very variable among historic as well as contemporary walleye [44,48]. Ichthyologists from the era of the “blue pike” reported a large numbers of intergrades in color, as well as among all morphological traits [44,48,49].

Some fishes, including lake trout, whitefish *Coregonus* spp., and Arctic char *Salvelinus alpinus* (Linnaeus 1758), have been regarded as multiple morphological races that developed through adaptation to northern proglacial lakes [106], but possess low genetic divergence [20]. The “blue pike” was reported to inhabit deeper waters, have slower growth [107], and a larger eye [42,this study] compared to walleye. Slower growth likewise characterizes walleye in eastern Lake Erie today [108]. We find that although “blue pike” and walleye display some slight morphological variation, this is rather negligible, and unaccompanied by population genetic distinction, rendering its subspecies status invalid. “Blue pike” were walleye, and fell within the normal range of walleye population variation.

The turquoise-colored mucus walleye from McKim Lake (site D) do not genetically differ from co-occurring yellow walleye. Stepien and Faber [41] likewise analyzed several assorted turquoise mucus walleye from a variety of Canadian Shield lakes using entire mtDNA control region sequences and found no genetic distinction from the normal variation of walleye. Paradis and Magnan [109] morphologically compared sympatric yellow and turquoise mucus walleye in five Canadian Shield lakes near Quebec, reporting longer head lengths and smaller interorbital distances in some of the latter. However, early fishery biologists found that these turquoise mucus walleye did not possess the morphological characteristics of “blue pike” [49]. Laporte et al. [110] alleged slight genetic difference between turquoise mucus and yellow walleye populations sampled within a lake using amplified fragment length polymorphism markers and assignment tests, but lacked diagnostic alleles and their genetic distance analyses showed no significant bootstrap support. It may be that there are some population-level variants within some lakes across the range of the Canadian Shield; many such distinctions among walleye spawning groups are found in the present study and others [30,39], but these do not warrant taxonomic recognition.

Occasional steel-blue colored walleye regularly are reported from Lake Erie and other waters, including the Ohio River drainage [44,48]. Yellow perch that are dark blue in color also co-occur [44]. We analyzed mtDNA control region from a steel-grey/blue walleye individual sampled in western Lake Erie near Sandusky OH and found it had mtDNA haplotype 1, the most common walleye haplotype. A skin scraping revealed no turquoise mucus. Wayne Schaeffer (pers. comm., University of Wisconsin, September 2013) also found no turquoise mucus or sandercyanin in Lake Erie walleye using the methods of Yu et al. [47]. Overall, no diagnosable genetic or morphological characters have been found that distinguish historic “blue pike” from walleye, rendering its subspecies status invalid.

### Effects of climate change on walleye populations

Global temperatures are predicted to increase over the next 50 years, with the Great Lakes region rising by 5–5.5°C, becoming more like today’s Gulf Coast [111]. Today, Lake Erie houses the largest walleye abundance [45] and greatest genetic diversity. Increased temperatures are predicted to shift walleye distribution northward [23]. Fringe populations may experience declines and increased isolation, with bottlenecks and drift reducing genetic variation, accompanied by loss of unique haplotypes and alleles. Hence, valuable genetic resources may disappear as unconnected populations become sequestered.

High connectivity in Great Lakes’ watersheds allows ample dispersal opportunities, which may homogenize gene pools of distinctive spawning groups as they move northward and mix, producing a Wahlund effect. Thus, climate change may lead to decline of divergence patterns from today’s walleye spawning groups. Walleye likely will remain abundant and adapt, but unique variants may be lost. Common alleles may increase in frequency, raising concerns for retaining adaptive potential, which should become a management priority. It might be possible to utilize unique warm-adapted variants to the south (e.g., the North River, Y) and southeast (Ohio/New Rivers, W–X) to aid future walleye populations.

## Conclusions

This study increases understanding of historic and contemporary walleye genetic diversity and divergence patterns via a genetic window approach. Population patterns have been shaped by climate change and drainage connections, with northern ones tracing to post-glacial recolonization. Genetic diversity and abundance of Great Lakes' walleye appear to have rebounded after drastic habitat changes and industrialization of the early 1900s through the 1970s. The next step is to identify adaptations that underlie these genetic diversity and divergence patterns, via genomics [112,113]. Such applied knowledge will aid efforts to sustain natural populations in the face of ongoing climate change and new anthropogenic stressors. Our goal is that the present results will aid future walleye diversity and adaptedness.

## Methods

### Sample collection for genetic analysis

Fin clips of 1125 contemporary spawning adult walleye were sampled at 23 spawning locations (labeled A–P, S–Y) across the native range during the spring spawning runs (Figure 1, Table 1, Additional file 10), avoiding sites with documented anthropogenic introductions. Samples were collected directly by federal, state, and provincial agencies according to their regulations and permits, and by Stepien’s Great Lakes Genetics/Genomics Laboratory (University of Toledo’s Lake Erie Center, OH) under Ohio Division of Wildlife permit #140160 (issued to CAS). Samples were labeled, immediately placed in 95% ethanol, and archived at the Great Lakes Genetics/Genomics Laboratory. Genetic comparisons were made to historic formalin-preserved museum specimens of Lake Erie walleye (1923–49, site Q, *N* = 20 for mtDNA, *N* = 31 for μsat) and historic “blue pike” paratype individuals (1923, R, *N* = 20, 25). The latter had been identified by Hubbs [42] and are housed at University of Michigan’s Museum of Zoology, while some of the walleye came from Ohio State University's Museum of Biological Diversity (see Additional file 11 for list of individuals used). We also evaluated contemporary yellow- (*N* = 10) versus turquoise-colored mucus (*N* = 10) walleye sympatric in McKim Lake, Ontario, Canada (site D, Figure 1).

### DNA extraction

DNA was extracted following standard protocols from previous studies [38,53]. To circumvent contamination, museum sample extractions were conducted in a separate clean laboratory using separate autoclaved materials. Gloves were changed at all stages and between samples. Pre-extraction, formalin fixed tissues and controls were soaked in 10 mL phosphate buffered saline on an orbital shaker for 48 hours, changing buffer after 24 hours. Historic walleye and “blue pike” samples were extracted at separate times, with negative (no tissue) and positive controls (Lake Erie Maumee River walleye #AZE31); the latter were handled after all solutions were added and tubes were closed.

### Mitochondrial DNA sequence data collection and analyses

MtDNA control region sequence data (733 bp) were collected from a representative subset from each of the 23 contemporary walleye spawning groups (*N =* 711*,* 11–25 per site; Table 1) used for the nuclear μsat loci. Methods for amplification and sequencing of the 733 bp control region segment followed Haponski and Stepien [53] using the primers LW1-F [78] and HN20 [114]. We additionally sequenced formalin fixed historic Lake Erie walleye (*N* = 20) and “blue pike” (*N* = 20). Since formalin fixation often shears DNA into smaller fragments [101], we designed additional internal primers to amplify the control region in overlapping segments: LW1-F [78] and *Sander*crIR (5’ - CATTCATACTATTTTCTTGC - 3’), *Sander*crIF (5’ - AGTACATACTCTGTTACC - 3’) with HN20 [114], *Sander*crIF with *Svi*crIR2 (5’ - GTGATTTCCACTATTTATGC - 3’), and *Svi*crIF (5’ - GCAAGAAAATAGTATGAATG - 3’) with HN20. Walleye haplotypes were referenced to Stepien and Faber [41], who sequenced the entire mtDNA control region (~1,086 base pairs (bp)). These were trimmed to 733 bp, omitting the 5’ repeat section, thereby retaining seven of their 14 haplotypes (our haplotypes 1–7 [GenBank:U90617, JX442946–52]).

Haplotype relationships were analyzed with evolutionary trees [50] via maximum likelihood in PhyML v3.0 [115] and Bayesian analyses in MrBayes v3.2.1 [116]. Corrected Akaike information criteria from jModeltest v2[117] selected the TPM3uf model [118], including invariant sites (I = 0.3910) and the gamma distribution (α = 0.2750). Trees were rooted to the sauger *S. canadensis* (Griffith and Smith 1834), which is the sister species of walleye [52]. A statistical parsimony haplotype network was constructed with TCS v1.21[54]. Analyses in common between the mtDNA and nuclear μsat data sets are below in “Population genetic data analyses”.

### Nuclear microsatellite data collection and analyses

We evaluated population genetic structure among 23 contemporary walleye spawning groups (1125 individuals; Table 1, Additional file 10), adding 259 individuals and six spawning sites to the 866 individuals and 17 locations previously analyzed for nine μsat loci (*Svi*2, 4, 6, 7, 17, 18, 33, L6, and L7) in our laboratory [30,38] (Additional file 11). Data were compared with museum specimens sampled from Lake Erie, including 31 walleye (1923–1949) and 25 “blue pike” (1923) (Additional file 11).

PCR amplifications followed previous studies [38,53], including a positive control and a negative control. Loci were run individually for the formalin material. *Svi*17 and L7 failed to amplify in historic samples and thus were dropped, leaving seven for analyses. Allele scores were manually verified [38,53].

Analyses followed Stepien et al. [30] and Haponski and Stepien [53]. Conformance to HWE expectations and linkage disequilibrium was tested in Genepop v4.0 [119] and significance levels adjusted with sequential Bonferroni correction [120]. Loci were tested for possible selection using *F*_ST_ outlier comparisons in Lositan [56] and examined for null alleles, scoring errors, or large allele dropout with Micro-Checker v2.2.3 [57]. If null alleles were indicated, their frequencies per population and locus were estimated and potential influence on *F*_ST_ values evaluated in FreeNA [58]. Number of alleles (*N*_A_), inbreeding (*F*_IS_), overall genetic deviation (*F*_IT_), and divergences (*F*_ST_) were calculated across loci and samples with *F*stat v2.9.3.2 [121,122].

Structure v2.3.4 [61] was employed to evaluate hypotheses of differentiation among population groups, for contemporary spawning groups from *K* = 1 (null hypothesis of panmixia) to *K* = 29 (sites A–P, S–Y) and contemporary versus historic walleye from *K* = 1 to *K* = 11 (nine contemporary Lakes Erie (O–P) and Ontario (S–U) spawning groups, historic walleye (Q), and “blue pike” (R)), with 250000 burn-in replicates followed by 1000000 generations. Optimal *K* was selected according to the Evanno et al. [123] method. Geneclass2 [59] assigned individuals to putative populations of origin for contemporary walleye, historic walleye, and “blue pike”, using 1000000 simulations. Colony v2.0.5.0 [55] was employed to test for possible kin relationships (full siblings) in samples.

### Population genetic data analyses with both data sets

Genetic diversity comparisons included haplotype diversity (*H*_D_) and number of haplotypes (*N*_H_) for sequence data in Arlequin v3.5.1.3 [124] and those for nuclear μsat loci were observed (*H*_O_) and expected (*H*_E_) heterozygosities in Genepop, and *F*_IS_, *N*_A_, and allelic richness (*A*_R_) in *F*stat. Significant differences were determined with ANOVA in R v2.15.2 [125], followed by Tukey’s *post hoc* tests [126]. Convert v1.31 [127] calculated number and proportion of private haplotypes (*N*_PH_) and alleles (*N*_PA_), i.e., those unique to a sample.

Patterns of genetic divergence were evaluated using unbiased *F*_ST_ estimates [128] in *F*stat and pairwise exact tests of differentiation (*χ*^2^) [129] in Genepop. Genetic divergence results were used to identify true populations or taxa, i.e., those with significantly divergent gene pools, to address the question of the identity of the “blue pike” and other possibly distinctive groups. We also tested genetic isolation by geographic distance (nearest waterways) in Genepop and identified genetic discontinuities across the range with Barrier v2.2 [26]. Population relationships additionally were analyzed with neighbor-joining trees [130] of Nei’s [131]*D* genetic distances in Phylip v3.68 [132] and 2000 bootstrap pseudo-replications [133]. The tree was rooted to sauger. Hierarchical partitioning of genetic variation was evaluated with AMOVA [60] in Arlequin, including: (a) between historic and modern samples, (b) among drainages, and (c) between lake and river spawners.

### Morphological comparisons

Morphological and meristic data were collected and compared between preserved historic Lake Erie walleye (*N* = 51 individuals) and “blue pike” samples (*N* = 52, including 30 paratypes; all individuals are listed in Additional file 11). We compared nine morphometric measurements and five meristic characters from Hubbs and Lagler [45], using identical standard protocols taken by a single investigator (AEH). Measurements (to 0.1 mm) included: standard, head, cheek, upper and lower jaw lengths, body depth, head and interorbital widths, and orbit diameter, with Mitutoyo vernier calipers (Aurora, IL). Measurements were taken to the bone of individual fish, to rule out influence of preservation technique. Meristic characters included: numbers of anal, first and second dorsal, pectoral, and pelvic fin rays, with a Leica Microsystems dissecting microscope (Buffalo Grove, IL). We also analyzed orbit: interorbital and upper: lower jaw ratios, reported by Trautman [44] to vary between walleye and “blue pike”.

Morphometric measurements first were standardized by removing size-dependent variation per Elliott et al. [134], and then log-transformed. Meristic data were not transformed; therefore morphometric and meristic data sets were analyzed separately, encompassing univariate and multivariate analyses in R. Each character was evaluated for departure from univariate normality with Shapiro-Francia tests [135]. Standard length, body depth, and orbit: interorbital ratio were the sole normally distributed characters. Their means were compared with Student’s *t*-tests, whereas Mann–Whitney *U* tests evaluated those that were not normally-distributed [126]. Nonparametric MANOVA [136] compared the overall difference between historic walleye and “blue pike”. Their overall morphological variation also was explored with PCA, using the covariance matrix. MANOVA and Wilks' lambda [126] tests evaluated among PCs, followed by individual ANOVAs.

DFA [137] compared historic walleye and “blue pike” in SAS v9.2 (SAS Institute Inc., Cary, NC). *χ*^2^ evaluated use of linear or quadratic DFA for significant covariance matrix heterogeneity, rejecting linear DFA (morphometric: *χ*^2^ = 103.216, df = 45, *p* < 0.001; meristic: *χ*^2^ = 32.253, df = 15, *p* = 0.006). Thus, quadratic DFA was used, which based each character on its own variance-covariance matrix, with leave-one-out jackknife re-sampling examining taxon assignment [138].

### Data set availability

The data sets supporting the results of this article are available in the Dryad digital repository [http://doi.org/10.5061/dryad.nk470] [139] and GenBank:U90617, JX442946–56, KC819843–54, and KF954732–35.

## Competing interests

The authors declare that they have no competing interests.

## Authors’ contributions

CAS conceived the project. AEH collected, analyzed, and interpreted the molecular and morphological data, drafted, and revised the paper. CAS oversaw the project design, assisted in data interpretation, added major parts and revisions to the paper, and extensively edited it. Both authors have read and approved the final manuscript.

## Authors’ information

This work formed part of the Ph.D. dissertation work of AEH in the laboratory of CAS, who now is a postdoctoral associate in the laboratory. CAS has worked on walleye genetic patterns for over two decades, and her Great Lakes Genetics/Genomics laboratory focuses on invasive species, as well as native fishes.

## Supplementary Material

Additional file 1 **Phylogenetic tree of contemporary and historic walleye mtDNA control region haplotypes.** Above branches = Bayesian posterior probabilities (pp) and Maximum likelihood bootstrap pseudoreplications. Nodes with ≥0.50 pp and ≥50% bootstrap support are reported. * = haplotypes found in historic walleye and “blue pike” samples; the latter had no unique haplotypes. All “blue pike” individuals had haplotype 3, which is one of the most common walleye haplotypes. Click here for file

Additional file 2 MtDNA control region haplotype frequencies for population samples, including 23 contemporary walleye spawning groups, historic Lake Erie walleye, and “blue pike”. Click here for file

Additional file 3 **Summarized genetic variation per microsatellite locus for population samples, including 23 contemporary walleye spawning groups, historic walleye, and “blue pike” samples, totaling 1181 individuals.** Table shows PCR annealing temperature (*T*_A_), number of alleles (*N*_A_), allelic size range (base pairs, bp), genetic deviation across all combined samples (*F*_IT_), mean genetic divergence among loci (*F*_ST_), inbreeding coefficient (*F*_IS_, average divergence within a spawning group), and neutrality test from the program Lositan [56]. Click here for file

Additional file 4 **Allelic sizes and distribution for seven nuclear μsat loci among samples, including: contemporary walleye from Lakes Erie (the western and eastern basins) and Ontario, historic Lake Erie walleye, and the “blue pike”.** Site labels (letters) match those in Table 1. Click here for file

Additional file 5 **Genetic isolation by geographic distance comparison among 23 contemporary spawning groups of walleye. A** mtDNA control region (*y* = 0.64*x*–3.67, *R*^2^ = 0.10, p < 0.001) and **B** seven nuclear μsat loci (*y* = 0.06*x*–0.29, *R*^2^ = 0.23, p = 0.005). Results from the seven loci are identical to those for nine loci (data not shown; see Stepien et al. [30]). Letters correspond to spawning group labels from Table 1. Click here for file

Additional file 6 **Pairwise exact tests of genetic differentiation among population samples (lettered) for control region sequence data (below diagonal) and seven nuclear μsat loci (above diagonal).** Results are congruent to *F*_ST_ comparisons and those from the seven nuclear μsat loci data are identical to values calculated based on nine loci (data not shown; also see Stepien et al. [30]), with difference at just the thousandth decimal place. Inf. = Infinite value denoted by Genepop [119], **bold** = significant following sequential Bonferroni corrections, *italics* = significant at α = 0.05, and normal text = not significant. Click here for file

Additional file 7 **Geneclass2 [**[59]**] assignments among contemporary walleye spawning groups.** Values = percentage assignment, parentheses = number of individuals assigning to that group, **bold** = self-assignment, and *italics* = greatest assignment. Numbers in parentheses next to spawning group denote sample size. Click here for file

Additional file 8 **Graph of *K *** **vs. *ΔK *** **based on Evanno et al. [**[123]**], showing the optimal *K *** **A contemporary walleye spawning groups and B contemporary Lakes Erie and Ontario and historic Lake Erie walleye (with “blue pike”).** Click here for file

Additional file 9 **Population genetic distance neighbor joining tree for contemporary walleye *Sander vitreus vitreus *spawning groups, historic walleye, and “blue pike” *S. v. “glaucus” *in relation to sauger *S. canadensis.*** Tree is based on combined frequencies of mtDNA control region haplotypes and alleles from the seven nuclear μsat loci. Click here for file

Additional file 10 **Latitude (Lat.) and longitude (Long.) for each contemporary walleye spawning group used in analyses.** Letters correspond to locations from Figure 1 and Table 1. Click here for file

Additional file 11 **Materials examined, including A museum specimens and B additional contemporary specimens that augmented prior work reported by Stepien et al. (2009, 2010, 2012).** Institutional abbreviations follow Leviton et al. [140]. Sample information is listed as follows: museum – lot number, number of samples, size range (SL, mm), locality, sampling date, and collector. Individuals that also were analyzed with molecular data are listed in the “Molecular Samples” section with their respective lot numbers and GenBank accession numbers. Individuals analyzed solely for molecular data are listed under “Molecular Samples” with lot number, number of samples, locality, sampling date, collector, and GenBank accession number. Click here for file

## References

[B1] Dawson TP Jackson ST House JI Prentice IC Mace GM Beyond predictions: biodiversity conservation in a changing climate Science 2011 332 53 58 21454781 10.1126/science.1200303

[B2] Hutchings JA Butchart SHM Collen B Schwartz MK Waples RS Red flags: correlates of impaired species recovery Trends Ecol Evol 2012 27 542 546 22784411 10.1016/j.tree.2012.06.005

[B3] Reed DH Frankham R Correlation between fitness and genetic diversity Conserv Biol 2003 17 230 237

[B4] Väli U Einarsson A Waits L Ellegren H To what extent do microsatellite markers reflect genome–wide genetic diversity in natural populations? Mol Ecol 2008 17 3808 3817 18647238 10.1111/j.1365-294X.2008.03876.x

[B5] Taubmann J Theissinger K Feldheim KA Laube I Graf W Haase P Johannesen J Pauls SU Modeling range shifts and assessing genetic diversity distribution of the montane aquatic mayfly *Ameletus inopinatus* in Europe under climate change scenarios Conserv Genet 2011 12 503 515

[B6] Allendorf FW Luikart G Aitken SN Conservation and the genetics of populations 2013 2 Oxford, UK: Blackwell Publishing

[B7] Davis MB Shaw RG Range shifts and adaptive responses to quaternary climate change Science 2001 292 673 679 11326089 10.1126/science.292.5517.673

[B8] Taylor EB Gow JL Witt J Zemlak R Connectivity among populations of pygmy whitefish (*Prosopium coulterii*) in northwestern North America inferred from microsatellite DNA analyses Can J Zool 2011 89 255 266

[B9] Carvalho GR Evolutionary aspects of fish distribution: genetic variability and adaptation J Fish Biol 1993 43 53 73

[B10] Ozerov MY Veselov AE Lumme J Primmer CR “Riverscape” genetics: river characteristics influence the genetic structure and diversity of anadromous and freshwater Atlantic salmon (*Salmo salar*) populations in northwest Russia Can J Fish Aquat Sci 2012 69 1947 1958

[B11] Slatkin M Gene flow in natural populations Annu Rev Ecol Syst 1985 16 393 430

[B12] Wofford JEB Gresswell RE Banks MA Influence of barriers to movement on within–watershed genetic variation of coastal cutthroat trout Ecol Appl 2005 15 628 637

[B13] Moran GF Hopper SD Genetic diversity and the insular population structure of the rare granite rock species, *Eucalyptus caesia* Benth Aust J Bot 1983 31 161 172

[B14] Coulon A Fitzpatrick JW Bowman R Lovette IJ Mind the gap: genetic distance increases with habitat gap size in Florida scrub jays Biol Lett 2012 8 582 585 22357936 10.1098/rsbl.2011.1244 PMC3391449

[B15] Poissant J Knight TW Ferguson MM Nonequilibrium conditions following landscape rearrangement: the relative contribution of past and current hydrological landscapes on the genetic structure of a stream–dwelling fish Mol Ecol 2005 14 1321 1331 15813773 10.1111/j.1365-294X.2005.02500.x

[B16] Huey JA Baker AM Hughes JM High levels of genetic structure in the Australian freshwater fish, *Ambassis macleayi* J N Am Benthol Soc 2010 29 1148 1160

[B17] Waples RS A multispecies approach to the analysis of gene flow in marine shore fishes Evolution 1987 41 385 400 10.1111/j.1558-5646.1987.tb05805.x 28568763

[B18] Pringle JM Wares JP Going against the flow: maintenance of alongshore variation in allele frequency in a coastal ocean Mar Ecol-Prog Ser 2007 335 69 84

[B19] Hewitt GM Some genetic consequences of ice ages, and their role in divergence and speciation Biol J Linn Soc 1996 58 247 276

[B20] Bernatchez L Wilson CC Comparative phylogeography of Nearctic and Palearctic fishes Mol Ecol 1998 7 431 452

[B21] Ward RD Billington N Hebert PDN Comparison of allozyme and mitochondrial DNA variation in populations of walleye, *Stizostedion vitreum* Can J Fish Aquat Sci 1989 46 2074 2084

[B22] Billington N Barrette RJ Hebert PDN Management implications of mitochondrial DNA variation in walleye stocks N Am J Fish Manage 1992 12 276 284

[B23] Chu C Mandrak NE Minns CK Potential impacts of climate change on the distributions of several common and rare freshwater fishes in Canada Divers Distrib 2005 11 299 310

[B24] Sharma S Jackson DA Minns CK Shuter BJ Will northern fish populations be in hot water because of climate change? Glob Change Biol 2007 13 2052 2064

[B25] Billington N Wilson C Sloss BL Barton BA Distribution and population genetics of walleye and sauger Biology, management, and culture of Walleye and Sauger 2011 Bethesda, MD: American Fisheries Society 105 132

[B26] Manni F Guérard E Heyer E Geographic patterns of (genetic, morphologic, linguistic) variation: how barriers can be detected by using Monmonier’s algorithm Hum Biol 2004 76 173 190 Available at http://www.mnhn.fr/mnhn/ecoanthropologie/software/barrier.html 15359530 10.1353/hub.2004.0034

[B27] Faulks LK Gilligan DM Beheregaray LB The role of anthropogenic vs. natural in–stream structures in determining connectivity and genetic diversity in an endangered freshwater fish, Macquarie perch (*Macquaria australasica*) Evol Appl 2011 4 589 601 10.1111/j.1752-4571.2011.00183.x PMC3352423 25568007

[B28] Williams JE Haak AL Neville HM Colyer WT Potential consequences of climate change to persistence of cutthroat trout populations N Am J Fish Manage 2009 29 533 548

[B29] Jonsson B Jonsson N A review of the likely effects of climate change on anadromous Atlantic salmon *Salmo salar* and brown trout *Salmo trutta,* with particular reference to water temperature and flow J Fish Biol 2009 75 2381 2447 20738500 10.1111/j.1095-8649.2009.02380.x

[B30] Stepien CA Murphy DJ Lohner RN Sepulveda–Villet OJ Haponski AE Signatures of vicariance, postglacial dispersal, and spawning philopatry: population genetics and biogeography of the walleye *Sander vitreus* Mol Ecol 2009 18 3411 3428 19659479 10.1111/j.1365-294X.2009.04291.x

[B31] Hauser L Adcock GJ Smith PJ Ramírez JHB Carvalho GR Loss of microsatellite diversity and low effective population size in an overexploited population of New Zealand snapper (*Pagrus auratus*) PNAS 2002 99 11742 11747 12185245 10.1073/pnas.172242899 PMC129339

[B32] Locke B Belore M Cook A Einhouse D Kenyon R Knight R Newman K Ryan P Wright E Walleye management plan Lake Erie Committee Great Lakes Fishery Commission 2005 Available at http://www.glfc.org/lakecom/lec/WTG_docs/other_reports_and_docs/wmp20051207.pdf

[B33] Nate NA Hansen MJ Rudstam LG Knight RL Newman SP Barton BA Population and community dynamics of walleye Biology, management, and culture of Walleye and Sauger 2011 Bethesda, MD: American Fisheries Society 321 374

[B34] Schmalz PJ Fayram AH Isermann DA Newman SP Edwards CJ Barton BA Harvest and exploitation Biology, management, and culture of Walleye and Sauger 2011 Bethesda, MD: American Fisheries Society 375 401

[B35] Barton BA Barry TP Barton BA Reproduction and environmental biology Biology, management, and culture of Walleye and Sauger 2011 Bethesda, MD: American Fisheries Society 199 231

[B36] Jennings MJ Claussen JE Philipp DP Evidence for heritable preferences for spawning habitat between two walleye populations T Am Fish Soc 1996 125 978 986

[B37] Bozek MA Baccante DA Lester NP Barton BA Walleye and sauger life history Biology, management, and culture of Walleye and Sauger 2011 Bethesda, MD: American Fisheries Society 233 301

[B38] Stepien CA Banda JA Murphy DJ Haponski AE Temporal and spatial genetic consistency of walleye (*Sander vitreus*) spawning groups T Am Fish Soc 2012 141 660 672

[B39] Strange RM Stepien CA Genetic divergence and connectivity among river and reef spawning groups of walleye (*Sander vitreus*) in Lake Erie Can J Fish Aquat Sci 2007 64 437 448

[B40] Stepien CA Murphy DJ Lohner RN Haponski AE Sepulveda–Villet OJ Roseman E, Kocovsky P, Vandergoot C Status and delineation of walleye (*Sander vitreus*) genetic stock structure across the Great Lakes Status of walleye in the Great Lakes: proceedings of the 2006 symposium 2010 Ann Arbor, MI: Great Lakes Fishery Commission Technical Report 69 189 223

[B41] Stepien CA Faber JE Population genetic structure, phylogeography, and spawning philopatry in walleye (*Stizostedion vitreum*) from mtDNA control region sequences Mol Ecol 1998 7 1757 1769 9859203 10.1046/j.1365-294x.1998.00512.x

[B42] Hubbs CL A check–list of the fishes of the Great Lakes and tributary waters, with nomenclatorial notes and analytical keys 1926 Ann Arbor, MI: University of Michigan Museum of Zoology Miscellaneous Publication 15

[B43] Stone FL A study of the taxonomy of the blue and yellow pikeperches (Stizostedion) of Lake Erie and Lake Ontario Unpublished Ph.D. dissertation 1948 Rochester, NY: University of Rochester

[B44] Trautman MB The fishes of Ohio 1981 Columbus, OH: Ohio State University Press

[B45] Hubbs CL Lagler KF Fishes of the Great Lakes Region. (G.R. Smith, revised) 2004 Ann Arbor, MI: University of Michigan

[B46] Noecker RJ Endangered species list revisions: a summary of delisting and downlisting CRS Report for Congress 98–32 ENR 1998

[B47] Yu C Ferraro D Ramaswamy S Schmitz MH Schaefer WF Gibson DT Purification and properties of sandercyanin, a blue protein secreted in the mucus of blue forms of walleye, *Sander vitreus* Environ Biol Fish 2008 82 51 58

[B48] Scott WB Crossman EJ Freshwater fishes of Canada Fish Res Board Can 1973 184 1 196

[B49] Campbell RR Status of the blue walleye, *Stizostedion vitreum glaucum*, in Canada Can Field Nat 1987 101 245 252

[B50] Avise JC Molecular markers, natural history, and evolution 2004 2 Sunderland, MA: Sinauer Associates

[B51] Wang IJ Recognizing the temporal distinctions between landscape genetics and phylogeography Mol Ecol 2010 19 2605 2608 20561197 10.1111/j.1365-294X.2010.04715.x

[B52] Haponski AE Stepien CA Phylogenetic and biogeographic relationships of the *Sander* pikeperches (Perciformes: Percidae): patterns across North America and Eurasia Biol J Linn Soc 2013 110 156 179

[B53] Haponski AE Stepien CA Genetic connectivity and diversity of walleye (*Sander vitreus*) spawning groups in the Huron–Erie Corridor J Great Lakes Res 2014 49 2014 89 100

[B54] Clement M Posada D Crandall KA TCS: a computer program to estimate gene genealogies Mol Ecol 2000 9 1657 1660 Available at http://darwin.uvigo.es/software/tcs.html 11050560 10.1046/j.1365-294x.2000.01020.x

[B55] Jones O Wang J COLONY: a program for parentage and sibship inference from multilocus genotype data Mol Ecol Resour 2009 10 551 555 Available at http://www.zsl.org/science/software/colony 21565056 10.1111/j.1755-0998.2009.02787.x

[B56] Antao T Lopes A Lopes RJ Beja–Pereira A Luikart G LOSITAN: a workbench to detect molecular adaptation based on a Fst–outlier method BMC Bioinformatics 2008 9 323 Available at http://popgen.net/soft/lositan/18662398 10.1186/1471-2105-9-323 PMC2515854

[B57] van Oosterhout C Hutchinson WF Wills DPM Shipley P Micro–checker: software for identifying and correcting genotyping errors in microsatellite data Mol Ecol Notes 2004 4 535 538 Available at http://www.microchecker.hull.ac.uk/

[B58] Chapuis M Estoup A Microsatellite null alleles and estimation of population differentiation Mol Biol Evol 2007 24 621 631 17150975 10.1093/molbev/msl191

[B59] Piry S Alapetite A Cornuet JM Paetkau D Baudouin L Estoup A Geneclass2: a software for genetic assignment and first–generation migrant detection J Hered 2004 95 536 539 Available at http://www.ensam.inra.fr/URLB/index.html 15475402 10.1093/jhered/esh074

[B60] Excoffier L Smouse P Quattro J Analysis of molecular variance inferred from metric distances among DNA haplotypes: application to human mitochondrial DNA restriction data Genetics 1992 131 479 491 1644282 10.1093/genetics/131.2.479 PMC1205020

[B61] Pritchard JK Stephens M Donnelly P Inference of population structure using multilocus genotype data Genetics 2000 155 945 959 Available at http://pritchardlab.stanford.edu/structure_software/release_versions/v2.3.4/html/WhatsNew.html 10835412 10.1093/genetics/155.2.945 PMC1461096

[B62] Palmer GC Culver C Dutton D Murphy BR Hallerman EM Billington N Williams J Genetic distinct walleye stocks in Claytor Lake and the Upper New River, Virginia P Southeast Fish Wild Agencies 2006 60 125 131

[B63] Danzmann RG Morgan RP Jones MW Bernatchez L Ihssen PH A major sextet of mitochondrial DNA phylogenetic assemblages extant in eastern North American brook trout (*Salvelinus fontinalis*): distribution and postglacial dispersal patterns Can J Fish Aquat Sci 1998 76 1300 1318

[B64] Stepien CA Murphy DJ Strange RM Broad- to fine-scale population genetic patterning in the smallmouth bass *Micropterus dolomieu* across the Laurentian Great Lakes and beyond: an interplay of behaviour and geography Mol Ecol 2007 16 1605 1624 17402977 10.1111/j.1365-294X.2006.03168.x

[B65] Haponski AE Stepien CA Molecular, morphological, and biogeographic resolution of cryptic taxa in the greenside darter *Etheostoma blennioides* complex Mol Phylogenet Evol 2008 49 69 83 18703148 10.1016/j.ympev.2008.07.013

[B66] Haponski AE Stepien CA Landscape genetic patterns of the rainbow darter: a watershed analysis of mitochondrial DNA sequences and nuclear microsatellites J Fish Biol 2009 75 2244 2268 20738685 10.1111/j.1095-8649.2009.02414.x

[B67] Sepulveda–Villet OJ Stepien CA Waterscape genetics of the yellow perch (*Perca flavescens*): patterns across large connected ecosystems and isolated relict populations Mol Ecol 2012 21 5795 5826 23078285 10.1111/mec.12044

[B68] Boschung HT Mayden RL Fishes of Alabama 2004 Washington D.C: Smithsonian Institution

[B69] Walter RP Cena CJ Morgan GE Heath DD Historical and anthropogenic factors affecting the population genetic structure of Ontario’s inland lake populations of walleye (*Sander vitreus*) J Hered 2012 103 831 841 23125407 10.1093/jhered/ess066

[B70] Karsiotis SI Sullivan TJ Stepien CA Population genetic structure and comparative diversity of smallmouth bass: patterns from two genomes J Fish Biol In review 10.1111/jfb.13296 28321848

[B71] Rawson MR Yellow perch movements Columbus, OH: Ohio Department of Natural Resources Job Program Report Dingell–Johnson Project Number F–35–R–18, Study Number. 4, July 1, 1979–June 30, 1980

[B72] Lyons J Kanehl P Philipp DP, Ridgway MS Seasonal movements of smallmouth bass in streams Black bass: ecology, conservation, and management 2002 Bethesda, MD: American Fisheries Society 149 160

[B73] Colby PJ McNicol RE Ryder RA Synopsis of biological data on the walleye Stizostedion v. vitreum 1979 Rome, IT: FAO Fisheries Synopsis 119

[B74] Gerlach G Schardt U Eckmann R Meyer A Kin–structured subpopulations in Eurasian perch (*Perca fluviatilis* L.) Heredity 2001 86 213 221 11380667 10.1046/j.1365-2540.2001.00825.x

[B75] Behrmann–Godel J Gerlach G First evidence for postzygotic reproductive isolation between two populations of Eurasian perch (*Perca fluviatilis* L.) within Lake Constance Front Zool 2008 5 1 7 18218080 10.1186/1742-9994-5-3 PMC2248191

[B76] Sullivan TJ Stepien CA Temporal population genetic structure of the yellow perch *Perca flavescens* (Percidae: Teleostei) within a complex lakescape T Am Fish Soc In review

[B77] Hewitt GM Speciation, hybrid zones and phylogeography - or seeing genes in space and time Mol Ecol 2001 10 537 549 11298967 10.1046/j.1365-294x.2001.01202.x

[B78] Gatt MH Fraser DJ Liskauskas AP Ferguson MM Mitochondrial DNA variation and stock structure of walleye from eastern Lake Huron: an analysis of contemporary and historical samples T Am Fish Soc 2002 131 99 108

[B79] Nesbø CL Fossheim T Vollestad LA Jakobsen KS Genetic divergence and phylogeographic relationships among European perch (*Perca fluviatilis*) populations reflect glacial refugia and postglacial colonization Mol Ecol 1999 8 1387 1404 10564445 10.1046/j.1365-294x.1999.00699.x

[B80] Stepien CA Dillon AK Chandler MD Genetic identity, phylogeography, and systematics of ruffe *Gymnocephalus* in the North American Great Lakes and Eurasia J Great Lakes Res 1998 24 361 378

[B81] Hartman WL The effects of exploitation, environmental changes, and new species on the fish habitats and resources of Lake Erie 1973 Ann Arbor, MI: Great Lakes Fishery Commission Technical Report 22

[B82] Hartig JH Zarull MA Ciborowski JJH Gannon JE Wilke E Norwood G Vincent AN Long–term ecosystem monitoring and assessment of the Detroit River and western Lake Erie Environ Monit Assess 2009 158 87 104 18850284 10.1007/s10661-008-0567-0

[B83] Bennion DH Manny BA Construction of shipping channels in the Detroit River-history and environmental consequences 2011 Reston, VA: U.S. Geological Survey Scientific Investigations Report 2011–5122

[B84] Ryan PA Knight R MacGregor R Towns G Hoopes R Culligan W Fish–community goals and objectives for Lake Erie 2003 Ann Arbor, MI: Great Lakes Fishery Commission Special Publication 03–02

[B85] Manny BA Kenaga D The Detroit River: effects of contaminants and human activities on aquatic plants and animals and their habitats Hydrobiologia 1991 219 269 279

[B86] Guinand B Scribner KT Page KS Burnham-Curtis MK Genetic variation over space and time: analyses of extinct and remnant lake trout populations in the Upper Great Lakes P R Soc London B 2003 270 425 433 10.1098/rspb.2002.2250 PMC1691259 12639323

[B87] Stott W Ebener MP Mohr L Hartman T Johnson J Roseman EF Spatial and temporal genetic diversity of lake whitefish (*Coregonus clupeaformis* (Mitchill)) from Lake Huron and Lake Erie Adv Limnol 2013 64 205 222

[B88] Hutchinson WF van Oosterhout C Rogers SI Carvalho GR Temporal analysis of archived samples indicates marked genetic changes in declining North Sea Cod (*Gadus morhua*) P R Soc London B 2003 270 2125 2132 10.1098/rspb.2003.2493 PMC1691486 14561275

[B89] Olson DE Scidmore WJ Homing behavior of spawning walleyes T Am Fish Soc 1962 91 355 361

[B90] Colby PJ Nepszy SJ Variation among stocks of walleye (*Stizostedion vitreum vitreum*): management implications Can J Fish Aquat Sci 1981 38 1814 1831

[B91] Haas RC Bryant WC Smith KD Nuhfer AJ Movement and harvest of fish in Lake St. Clair, St. Clair River, and Detroit River 1985 Final Report Winter Navigation Study U.S. Army Corps of Engineers: Detroit, MI

[B92] Wang HY Rutherford ES Cook HA Einhouse DW Haas RC Johnson TB Kenyon R Locke B Turner MW Movement of walleye in Lakes Erie and St. Clair inferred from tag return and fisheries data T Am Fish Soc 2007 136 539 551

[B93] Haas RC Thomas MV Hartig JH, Zarull MA, Ciborowski JJH, Gannon JE, Wilke E, Norwood G, Vincent A The walleye population of Lake Erie State of the strait: status and trends of key indicators 2007 Windsor, ON: Great Lakes Institute for Environmental Research Occasional Publication No. 5 226 229

[B94] WTG (Walleye Task Group of the Lake Erie Committee, Great Lakes Fishery Commission) Report for 2012 by the Lake Erie Walleye task group 2013 Ann Arbor, MI: Great Lakes Fishery Commission Available at http://www.glfc.org/lakecom/lec/WTG_docs/annual_reports/WTG_report_2013.pdf

[B95] Franckowiak RP Sloss BL Bozek MA Newman SP Temporal effective size estimates of a managed walleye *Sander vitreus* population and implications for genetic–based management J Fish Biol 2009 74 1086 1103 20735621 10.1111/j.1095-8649.2008.02170.x

[B96] Garner SW Bobrowicz SM Wilson CC Genetic and ecological assessment of population rehabilitation: Walleye in Lake Superior Ecol Appl 2013 23 594 605 23734488 10.1890/12-1099.1

[B97] Waples RS Conservation genetics of Pacific salmon. III. Estimating effective population size J Hered 1990 81 277 289

[B98] Nielsen EE Hansen MM Waking the dead: the value of population genetic analyses of historical samples Fish Fish 2008 9 450 461

[B99] Taberlet P Griffin S Goossens B Questiau S Manceau V Escaravage N Waits LP Bouvet J Reliable genotyping of samples with very low DNA quantities using PCR Nucleic Acids Res 1996 24 3189 3194 8774899 10.1093/nar/24.16.3189 PMC146079

[B100] DeWoody J Nason JD Hipkins VD Mitigating scoring errors in microsatellite data from wild populations Mol Ecol Notes 2006 6 951 957

[B101] Shedlock AM Haygood MG Pietsch TW Bentz P Enhanced DNA extraction and PCR amplification of mitochondrial genes from formalin fixed museum specimens Biotechniques 1997 22 394 400 9067006 10.2144/97223bm03

[B102] Ugelvig LV Nielsen PS Boomsma JJ Nash DR Reconstructing eight decades of genetic variation in an isolated Danish population of the large blue butterfly *Maculinea arion* BMC Evol Biol 2011 11 201 21745368 10.1186/1471-2148-11-201 PMC3146443

[B103] Wiley EO Mayden RL Wheeler QD, Meier R The Evolutionary Species Concept Species concepts and phylogenetic theory, a debate 2000 New York, NY: Columbia University Press 70 89

[B104] Mishler BD Theriot EC Wheeler QD, Meier R The phylogenetic species concept (sensu Mishler and Theriot): Monophyly, apomorphy, and phylogenetic species concepts Species concepts and phylogenetic theory, a debate 2000 New York, NY: Columbia University Press 44 54

[B105] Hebert PDN Penton EH Burns JM Danzen DH Hallwachs W Ten species in one: DNA barcoding reveals cryptic species in the neotropical skipper butterfly *Astraptes fulgerator* PNAS 2004 101 14812 14817 15465915 10.1073/pnas.0406166101 PMC522015

[B106] Robinson BW Parsons KJ Changing times, spaces, and faces: tests and implications of adaptive morphological plasticity in the fishes of northern postglacial lakes Can J Fish Aquat Sci 2002 59 1819 1833

[B107] Parsons JW Contributions of year–classes of blue pike to the commercial fishery of Lake Erie, 1943–59 Fish Res Board Can 1967 24 1035 1066

[B108] Einhouse DW MacDougall TM Roseman E, Kocovsky P, Vandergoot C An emerging view of the mixed-stock structure of Lake Erie’s eastern-basin walleye population Status of walleye in the Great Lakes: proceedings of the 2006 symposium 2010 Ann Arbor, MI: Great Lakes Fishery Commission Technical Report 69 151 164

[B109] Paradis Y Magnan P Phenotypic variation of walleye, *Sander vitreus,* in Canadian Shield lakes: new insights on percid polymorphism Env Biol Fish 2005 73 357 366

[B110] Laporte M Magnan P Angers B Genetic differentiation between the blue and the yellow phenotypes of walleye (*Sander vitreus*): an example of parallel evolution Ecoscience 2011 18 124 129

[B111] Hayhoe K VanDorn J Croley T Schlegal N Wuebbles D Regional climate change projections for Chicago and the US Great Lakes J Great Lakes Res 2010 36 7 21

[B112] Allendorf FW Hohenlohe PA Luikart G Genomics and the future of conservation genetics Nat Rev Genet 2010 11 697 710 20847747 10.1038/nrg2844

[B113] Bradbury IR Hubert S Higgins B Bowman S Borza T Paterson IG Snelgrove PVR Morris CJ Gregory RS Hardie D Hutchings JA Ruzzante DE Taggart CT Bentzen P Genomic islands of divergence and their consequences for the resolution of spatial structure in an exploited marine fish Evol Appl 2013 6 450 461 23745137 10.1111/eva.12026 PMC3673473

[B114] Bernatchez L Danzmann RG Congruence in control-region sequence and restriction-site variation in mitochondrial DNA of brook charr (*Salvelinus fontinalis* Mitchill) Mol Biol Evol 1993 10 1002 1014

[B115] Guindon S Dufayard JF Lefort V Anisimova M Hordijk W Gascuel O New Algorithms and methods to estimate maximum–likelihood phylogenies: assessing the performance of PhyML 3.0 Systematic Biol 2010 59 307 321 Available at http://www.atgc-montpellier.fr/phyml/10.1093/sysbio/syq010 20525638

[B116] Ronquist F Huelsenbeck JP MrBayes 3: Bayesian phylogenetic inference under mixed models Bioinformatics 2003 19 1572 1574 http://mrbayes.csit.fsu.edu/(v3.1.2, 2005) 12912839 10.1093/bioinformatics/btg180

[B117] Darriba D Taboada GL Doallom R Posada D jModelTest 2: more models, new heuristics and parallel computing Nat Methods 2012 9 772 Available at http://code.google.com/p/jmodeltest2/22847109 10.1038/nmeth.2109 PMC4594756

[B118] Posada D jModelTest: phylogenetic model averaging Mol Biol Evol 2008 25 1253 1256 Available at http://code.google.com/p/jmodeltest2/18397919 10.1093/molbev/msn083

[B119] Rousset F Genepop’008: a complete re–implementation of the Genepop software for Windows and Linux Mol Ecol Resour 2008 8 103 106 Available at http://kimura.univ-montp2.fr/~rousset/Genepop.htm 21585727 10.1111/j.1471-8286.2007.01931.x

[B120] Rice RM Analyzing tables of statistical tests Evolution 1989 43 223 225 10.1111/j.1558-5646.1989.tb04220.x 28568501

[B121] Goudet J *F*stat version 1.2: a computer program to calculate *F*statistics J Hered 1995 86 485 486

[B122] Goudet J Fstat version 2.9.3.2 2002 Available at http://www2.unil.ch/popgen/softwares/fstat.htm

[B123] Evanno G Regnaut S Goudet J Detecting the number of clusters of individuals using the software structure: a simulation study Mol Ecol 2005 14 2611 2620 15969739 10.1111/j.1365-294X.2005.02553.x

[B124] Excoffier L Lischer HE Arlequin suite ver 3.5: a new series of programs to perform population genetics analyses under Linux and Windows Mol Ecol Resour 2010 10 564 567 Available at http://cmpg.unibe.ch/software/arlequin35/21565059 10.1111/j.1755-0998.2010.02847.x

[B125] R Development Core Team R: a language and environment for statistical computing 2012 Vienna, IT: R Foundation for Statistical Computing Available at http://www.R-project.org

[B126] Zar JH Biostatistical analysis 1999 4 Upper Saddle River, NJ: Prentice Hall

[B127] Glaubitz JC CONVERT: a user–friendly program to reformat diploid genotypic data for commonly used population genetic software packages Mol Ecol Notes 2004 4 309 310 http://www.agriculture.purdue.edu/fnr/html/faculty/rhodes/students%20and%20staff/glaubitz/software.htm

[B128] Weir BS Cockerham CC Estimating *F*–statistics for the analysis of population structure Evolution 1984 38 1358 1370 10.1111/j.1558-5646.1984.tb05657.x 28563791

[B129] Raymond M Rousset F An exact test for population differentiation Evolution 1995 49 1280 1283 10.1111/j.1558-5646.1995.tb04456.x 28568523

[B130] Saitou N Nei M The neighbor joining method: a new method for reconstructing phylogenetic trees Mol Biol Evol 1987 4 406 425 3447015 10.1093/oxfordjournals.molbev.a040454

[B131] Nei M Genetic distance between populations Am Nat 1972 106 283 282

[B132] Felsenstein J Phylip (Phylogeny Inference Package) version 3.6. Distributed by the author 2005 Seattle: Department of Genome Sciences, University of Washington http://evolution.genetics.washington.edu/phylip.html

[B133] Felsenstein J Confidence limits on phylogenies: an approach using the bootstrap Evolution 1985 39 783 791 10.1111/j.1558-5646.1985.tb00420.x 28561359

[B134] Elliott NG Haskard K Koslow JA Morphometric analysis of orange roughy (*Hoplostethus atlanticus*) off the continental slope of southern Australia J Fish Biol 1995 46 202 220

[B135] Royston P A pocket-calculator algorithm for the Shapiro-Francia test for non-normality: an application to medicine Stat Med 1993 12 181 184 8446812 10.1002/sim.4780120209

[B136] Anderson MJ A new method for non-parametric multivariate analysis of variance Austral Ecol 2001 26 32 46

[B137] dos Reis SF Pessôa LM Strauss RE Application of size–free canonical discriminant analysis to studies of geographic differentiation Braz J Genet 1990 13 509 520

[B138] McGarigal K Cushman S Stafford S Multivariate statistics for wildlife and ecology research 2000 New York, NY: Springer–Verlag

[B139] Haponski AE Stepien CA Nuclear DNA microsatellite loci and mitochondrial DNA sequence data from walleye Sander vitreus. Dryad 2014 http://doi.org/10.5061/dryad.nk470

[B140] Leviton AE Standards in herpetology and ichthyology, part 1. Standard symbolic codes for institutional resource collections in herpetology and ichthyology Copeia 1985 1985 802 832

