# Supplementary material for: A population genetic window into the past and future of the walleye Sander vitreus: relation to historic walleye and the extinct “blue pike” S. v. “glaucus”
Source: BMC Evol Biol. 2014 Jun 17;14:133. doi: 10.1186/1471-2148-14-133 (PMC4229939; doi:10.1186/1471-2148-14-133)
Supplement: Additional file 1 — Phylogenetic tree of contemporary and historic walleye mtDNA control region haplotypes. Above branches = Bayesian posterior probabilities (pp) and Maximum likelihood bootstrap pseudoreplications. Nodes with ≥0.50 pp and ≥50% bootstrap support are reported. * = haplotypes found in historic walleye and “blue pike” samples; the latter had no unique haplotypes. All “blue pike” individuals had haplotype 3, which is one of the most common walleye haplotypes. [file 1471-2148-14-133-S1.doc]

**Additional file 3**

**Summarized genetic variation per microsatellite locus for population samples, including 23 contemporary walleye spawning groups, historic walleye, and “blue pike” samples, totaling 1181 individuals**. Table shows PCR annealing temperature (*T*_A_), number of alleles (*N*_A_), allelic size range (base pairs, bp), genetic deviation across all combined samples (*F*_IT_), mean genetic divergence among loci (*F*_ST_), inbreeding coefficient (*F*_IS_, average divergence within a spawning group), and neutrality test from the program Lositan [53].

| **Locus** | **Source** | ***T*_A_ (ºC)** | ***N*_A_** | **Size range** | ***F*_IT_** | ***F*_ST_** | ***F*_IS_** | **Selection** |
| --- | --- | --- | --- | --- | --- | --- | --- | --- |
| *Svi*4 | 138 | 60 | 12 | 98−122 | 0.141 | 0.110 | 0.034 | Neutral |
| *Svi*6 | “ | 60 | 22 | 126−172 | 0.116 | 0.064 | 0.055 | Neutral |
| *Svi*17 | “ | 54 | 11 | 100−120 | 0.117 | 0.132 | –0.016 | Positive |
| *Svi*18 | “ | 65 | 8 | 114−128 | 0.200 | 0.113 | 0.098 | Neutral |
| *Svi*33 | “ | 60 | 16 | 72−106 | 0.082 | 0.064 | 0.019 | Neutral |
| *Svi*L6 | 139 | 54 | 22 | 92−140 | 0.061 | 0.067 | –0.007 | Neutral |
| *Svi*L7 | “ | 54 | 27 | 160−238 | 0.076 | 0.029 | 0.049 | Balancing |
| *Svi*2 | 140 | 60 | 17 | 178−222 | 0.069 | 0.064 | 0.005 | Neutral |
| *Svi*7 | “ | 60 | 20 | 140−198 | 0.167 | 0.110 | 0.065 | Neutral |
| Total | --- | --- | 155 | --- | 0.118 | 0.084 | 0.037 | --- |
