# Supplementary material for: A population genetic window into the past and future of the walleye Sander vitreus: relation to historic walleye and the extinct “blue pike” S. v. “glaucus”
Source: BMC Evol Biol. 2014 Jun 17;14:133. doi: 10.1186/1471-2148-14-133 (PMC4229939; doi:10.1186/1471-2148-14-133)
Supplement: Additional file 2 — MtDNA control region haplotype frequencies for population samples, including 23 contemporary walleye spawning groups, historic Lake Erie walleye, and “blue pike”. [file 1471-2148-14-133-S2.doc]

**Additional file 4**

**Allelic sizes and distribution for seven nuclear μsat loci among samples, including: contemporary walleye from Lakes Erie (the western and eastern basins) and Ontario, historic Lake Erie walleye, and the “blue pike”.** Site labels (letters) match those in Table 1.

**A *Svi***33

| **Allele length** | **O. Western basin** | **P. Eastern basin** | **Q. Historic walleye** | **R. “Blue pike”** | **S. Pigeon L.** | **T. Bay of Quinte** |
| --- | --- | --- | --- | --- | --- | --- |
| 72 | – | – | 0.02 | – | – | – |
| 80 | – | – | 0.07 | – | – | – |
| 82 | 0.01 | 0.01 | – | – | – | – |
| 84 | 0.06 | 0.08 | 0.04 | – | 0.10 | 0.07 |
| 86 | 0.13 | 0.12 | 0.41 | 0.48 | 0.14 | 0.17 |
| 88 | 0.14 | 0.10 | 0.02 | – | 0.09 | 0.19 |
| 90 | 0.05 | 0.03 | – | – | 0.02 | 0.04 |
| 92 | 0.04 | 0.06 | – | – | 0.16 | 0.06 |
| 94 | 0.19 | 0.18 | 0.43 | 0.52 | 0.04 | 0.06 |
| 96 | 0.26 | 0.22 | 0.02 | – | 0.19 | 0.14 |
| 98 | 0.03 | 0.06 | – | – | – | 0.01 |
| 100 | 0.09 | 0.13 | – | – | 0.24 | 0.22 |
| 102 | 0.02 | 0.02 | – | – | 0.03 | 0.03 |
| 104 | – | – | – | – | – | 0.01 |
| 106 | – | 0.01 | – | – | – | – |

**B *Svi***4

| **Allele length** | **O. Western basin** | **P. Eastern basin** | **Q. Historic walleye** | **R. “Blue pike”** | **S. Pigeon L.** | **T. Bay of Quinte** |
| --- | --- | --- | --- | --- | --- | --- |
| 98 | – | – | 0.02 | – | – | – |
| 104 | – | 0.03 | – | – | – | – |
| 106 | 0.06 | 0.11 | 0.02 | – | 0.02 | 0.02 |
| 108 | 0.01 | 0.03 | – | – | – | – |
| 110 | 0.18 | 0.18 | – | – | 0.03 | 0.09 |
| 112 | 0.03 | 0.04 | 0.06 | 0.02 | 0.05 | 0.03 |
| 114 | 0.15 | 0.15 | 0.43 | 0.57 | 0.17 | 0.24 |
| 116 | 0.40 | 0.32 | 0.48 | 0.41 | 0.66 | 0.48 |
| 118 | 0.17 | 0.13 | – | – | 0.07 | 0.14 |
| 120 | 0.01 | 0.01 | – | – | – | – |
| 122 | 0.01 | – | – | – | – | – |

**C *Svi***18

| **Allele length** | **O. Western basin** | **P. Eastern basin** | **Q. Historic walleye** | **R. “Blue pike”** | **S. Pigeon L.** | **T. Bay of Quinte** |
| --- | --- | --- | --- | --- | --- | --- |
| 114 | 0.01 | – | – | 0.02 | – | – |
| 116 | – | – | 0.05 | – | – | – |
| 118 | 0.223 | 0.19 | 0.85 | 0.50 | 0.52 | 0.38 |
| 120 | 0.02 | 0.04 | 0.03 | 0.44 | – | 0.01 |
| 122 | 0.23 | 0.29 | 0.03 | 0.02 | 0.16 | 0.13 |
| 124 | 0.50 | 0.47 | 0.03 | 0.02 | 0.22 | 0.46 |
| 126 | 0.02 | 0.01 | – | – | 0.10 | 0.02 |

**D *Svi***L6

| **Allele length** | **O. Western basin** | **P. Eastern basin** | **Q. Historic walleye** | **R. “Blue pike”** | **S. Pigeon L.** | **T. Bay of Quinte** |
| --- | --- | --- | --- | --- | --- | --- |
| 92 | – | – | 0.04 | – | – | – |
| 98 | – | – | 0.02 | – | – | – |
| 102 | – | – | 0.02 | – | – | – |
| 106 | 0.01 | – | – | – | – | – |
| 108 | 0.13 | 0.15 | 0.37 | 0.50 | 0.05 | 0.06 |
| 110 | 0.46 | 0.36 | 0.46 | 0.44 | 0.57 | 0.56 |
| 112 | 0.03 | 0.02 | – | 0.06 | – | 0.02 |
| 114 | – | 0.01 | 0.04 | – | – | – |
| 116 | 0.01 | – | – | – | – | 0.02 |
| 118 | – | – | – | – | – | 0.02 |
| 120 | – | – | – | – | – | 0.01 |
| 122 | 0.05 | 0.08 | – | – | 0.14 | 0.10 |
| 124 | 0.10 | 0.16 | 0.02 | – | 0.07 | 0.09 |
| 126 | 0.04 | 0.02 | – | – | – | – |
| 128 | 0.09 | 0.05 | 0.02 | – | – | 0.05 |
| 130 | 0.06 | 0.07 | 0.02 | – | 0.05 | 0.01 |
| 132 | 0.02 | 0.06 | – | – | 0.02 | 0.01 |
| 134 | 0.01 | 0.02 | – | – | 0.03 | 0.05 |
| 136 | 0.01 | 0.01 | – | – | 0.02 | – |
| 138 | – | 0.01 | – | – | 0.02 | – |
| 140 | – | – | – | – | 0.02 | – |

**E *Svi***2

| **Allele length** | **O. Western basin** | **P. Eastern basin** | **Q. Historic walleye** | **R. “Blue pike”** | **S. Pigeon L.** | **T. Bay of Quinte** |
| --- | --- | --- | --- | --- | --- | --- |
| 188 | 0.01 | – | – | – | – | – |
| 190 | 0.15 | 0.11 | – | – | 0.04 | 0.07 |
| 192 | 0.46 | 0.48 | 0.52 | 0.57 | 0.18 | 0.26 |
| 194 | 0.14 | 0.08 | 0.02 | – | 0.20 | 0.21 |
| 196 | 0.09 | 0.13 | 0.02 | – | 0.13 | 0.15 |
| 198 | 0.03 | 0.04 | – | – | 0.25 | 0.13 |
| 200 | 0.01 | 0.01 | 0.02 | 0.02 | 0.02 | 0.01 |
| 202 | 0.08 | 0.12 | 0.41 | 0.41 | 0.16 | 0.13 |
| 204 | 0.01 | 0.01 | – | – | – | 0.01 |
| 216 | 0.01 | 0.01 | – | – | – | 0.02 |
| 218 | – | 0.01 | – | – | 0.04 | 0.01 |
| 220 | 0.01 | 0.01 | – | – | – | – |
| 222 | 0.01 | – | – | – | – | – |

**F *Svi***6

| **Allele length** | **O. Western basin** | **P. Eastern basin** | **Q. Historic walleye** | **R. “Blue pike”** | **S. Pigeon L.** | **T. Bay of Quinte** |
| --- | --- | --- | --- | --- | --- | --- |
| 126 | 0.01 | – | – | – | – | – |
| 132 | – | 0.01 | 0.04 | – | – | – |
| 138 | – | – | 0.02 | – | – | – |
| 140 | 0.54 | 0.40 | 0.90 | 0.98 | 0.41 | 0.53 |
| 142 | 0.03 | 0.04 | – | 0.02 | 0.09 | 0.12 |
| 144 | 0.06 | 0.07 | – | – | 0.02 | 0.03 |
| 146 | 0.10 | 0.13 | 0.02 | – | 0.02 | 0.02 |
| 148 | 0.03 | 0.05 | – | – | 0.02 | 0.06 |
| 150 | 0.02 | 0.02 | – | – | 0.07 | 0.06 |
| 152 | 0.01 | 0.01 | – | – | – | 0.05 |
| 154 | 0.04 | 0.08 | – | – | 0.19 | 0.04 |
| 156 | 0.06 | 0.07 | 0.02 | – | 0.02 | 0.01 |
| 158 | 0.01 | – | – | – | – | 0.01 |
| 160 | 0.03 | 0.04 | – | – | 0.03 | 0.03 |
| 162 | 0.01 | 0.05 | – | – | 0.03 | – |
| 164 | 0.02 | 0.01 | – | – | 0.02 | 0.02 |
| 166 | 0.02 | 0.03 | – | – | 0.05 | 0.01 |
| 168 | 0.01 | – | – | – | 0.03 | 0.01 |

**G *Svi***7

| **Allele length** | **O. Western basin** | **P. Eastern basin** | **Q. Historic walleye** | **R. “Blue pike”** | **S. Pigeon L.** | **T. Bay of Quinte** |
| --- | --- | --- | --- | --- | --- | --- |
| 154 | 0.01 | 0.01 | – | – | – | 0.01 |
| 156 | 0.21 | 0.26 | 0.02 | – | 0.29 | 0.23 |
| 158 | 0.06 | 0.03 | 0.26 | 0.40 | 0.12 | 0.11 |
| 160 | 0.01 | 0.02 | 0.12 | – | – | – |
| 162 | 0.50 | 0.43 | 0.16 | – | 0.43 | 0.49 |
| 164 | 0.11 | 0.11 | 0.33 | 0.60 | 0.07 | 0.08 |
| 166 | 0.08 | 0.14 | 0.09 | – | 0.09 | 0.06 |
| 168 | 0.01 | – | – | – | – | – |
| 170 | 0.01 | – | – | – | – | – |
| 172 | 0.02 | 0.01 | – | – | – | – |
| 174 | – | 0.01 | – | – | – | – |
| 176 | 0.01 | 0.01 | – | – | – | 0.02 |
| 190 | – | – | 0.02 | – | – | – |
| 192 | – | – | 0.02 | – | – | – |
