# Supplementary material for: A population genetic window into the past and future of the walleye Sander vitreus: relation to historic walleye and the extinct “blue pike” S. v. “glaucus”
Source: BMC Evol Biol. 2014 Jun 17;14:133. doi: 10.1186/1471-2148-14-133 (PMC4229939; doi:10.1186/1471-2148-14-133)
Supplement: Additional file 3 — Summarized genetic variation per microsatellite locus for population samples, including 23 contemporary walleye spawning groups, historic walleye, and “blue pike” samples, totaling 1181 individuals. Table shows PCR annealing temperature (TA), number of alleles (NA), allelic size range (base pairs, bp), genetic deviation across all combined samples (FIT), mean genetic divergence among loci (FST), inbreeding coefficient (FIS, average divergence within a spawning group), and neutrality test from the program Lositan [56]. [file 1471-2148-14-133-S3.doc]

**
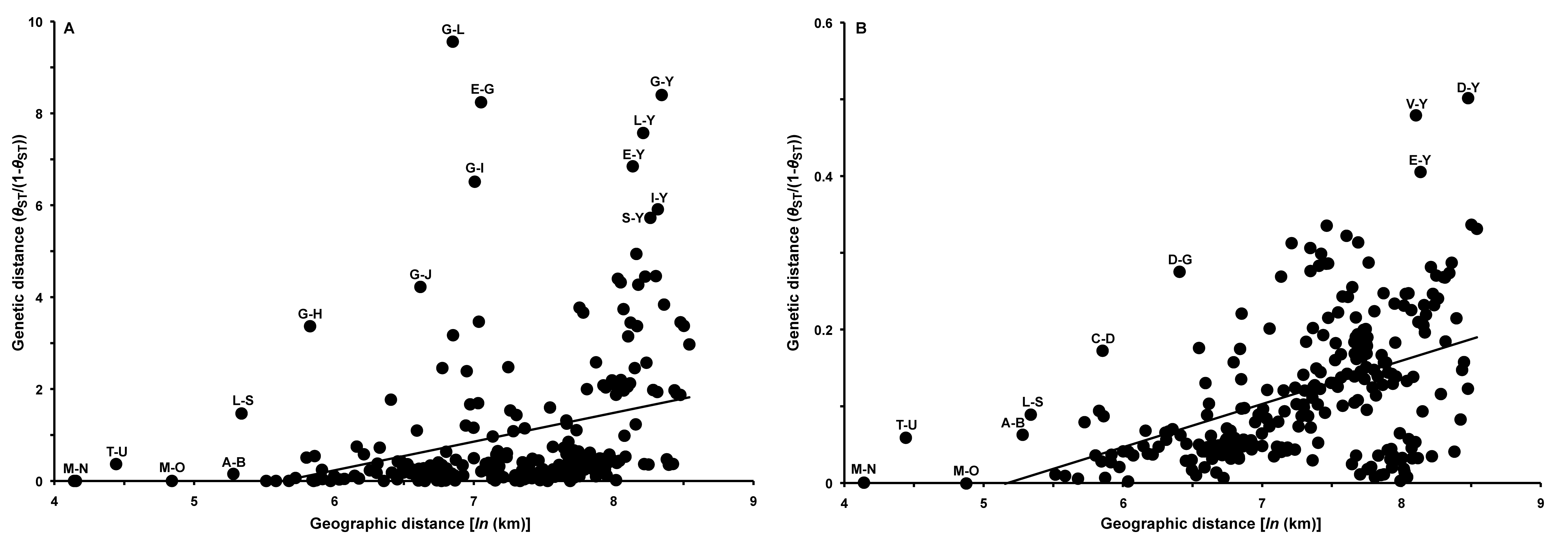
**

**Additional file 5**

**Genetic isolation by geographic distance comparison among 23 contemporary spawning groups of walleye**. **A** mtDNA control region (*y*=0.64*x*–3.67, *R*^2^=0.10, p<0.001) and **B** seven nuclear μsat loci (*y*=0.06*x*–0.29, *R*^2^=0.23, p=0.005). Results from the seven loci are identical to those for nine loci (data not shown; see Stepien et al. [28]). Letters correspond to spawning group labels from Table 1.
