# Supplementary material for: A population genetic window into the past and future of the walleye Sander vitreus: relation to historic walleye and the extinct “blue pike” S. v. “glaucus”
Source: BMC Evol Biol. 2014 Jun 17;14:133. doi: 10.1186/1471-2148-14-133 (PMC4229939; doi:10.1186/1471-2148-14-133)
Supplement: Additional file 4 — Allelic sizes and distribution for seven nuclear μsat loci among samples, including: contemporary walleye from Lakes Erie (the western and eastern basins) and Ontario, historic Lake Erie walleye, and the “blue pike”. Site labels (letters) match those in Table 1. [file 1471-2148-14-133-S4.doc]

**Additional file 6**

**Pairwise exact tests of genetic differentiation among population samples (lettered) for control region sequence data (below diagonal) and seven nuclear μsat loci (above diagonal).** Results are congruent to *F*_ST_ comparisons and those from the seven nuclear μsat loci data are identical to values calculated based on nine loci (data not shown; also see Stepien et al. [28]), with difference at just the thousandth decimal place. Inf.=Infinite value denoted by Genepop [119], **bold**=significant following sequential Bonferroni corrections, *italics*=significant at α=0.05, and normal text=not significant.

| **Location** | **A.** | **B.** | **C.** | **D.** | **E.** | **F.** | **G.** | **H.** | **I.** | **J.** | **K.** | **L.** | **M.** | **N.** | **O.** | **P.** | **Q.** | **R.** | **S.** | **T.** | **U.** | **V.** | **W.** | **X.** | **Y.** |
| --- | --- | --- | --- | --- | --- | --- | --- | --- | --- | --- | --- | --- | --- | --- | --- | --- | --- | --- | --- | --- | --- | --- | --- | --- | --- |
| A. Cedar L. | --- | **Inf.** | **70.05** | **Inf.** | **Inf.** | **Inf.** | **Inf.** | **Inf.** | **Inf.** | **Inf.** | **Inf.** | **Inf.** | **Inf.** | **Inf.** | **Inf.** | **Inf.** | **Inf.** | **Inf.** | **Inf.** | **Inf.** | **Inf.** | **Inf.** | **57.66** | **Inf.** | **Inf.** |
| B. L. Winnipeg | **17.20** | --- | **36.31** | **Inf.** | **Inf.** | **Inf.** | **53.29** | **Inf.** | **Inf.** | **Inf.** | **Inf.** | **Inf.** | **Inf.** | **Inf.** | **Inf.** | **Inf.** | **Inf.** | **Inf.** | **Inf.** | **Inf.** | **Inf.** | **Inf.** | **44.06** | **Inf.** | **Inf.** |
| C. L. of the Woods | **18.47** | *11.20* | --- | **Inf.** | **Inf.** | **Inf.** | **59.57** | **Inf.** | **Inf.** | **Inf.** | **Inf.** | **Inf.** | **Inf.** | **Inf.** | **Inf.** | **Inf.** | **Inf.** | **Inf.** | **Inf.** | **Inf.** | **Inf.** | **Inf.** | **47.65** | **Inf.** | **Inf.** |
| D. McKim L. | *6.37* | *14.61* | 5.88 | --- | **Inf.** | **Inf.** | **Inf.** | **Inf.** | **Inf.** | **Inf.** | **Inf.** | **Inf.** | **Inf.** | **Inf.** | **Inf.** | **Inf.** | **Inf.** | **Inf.** | **Inf.** | **Inf.** | **Inf.** | **Inf.** | **Inf.** | **Inf.** | **Inf.** |
| E. Mille Lacs | **21.55** | **Inf.** | *5.99* | *9.29* | --- | **Inf.** | **Inf.** | **Inf.** | **Inf.** | **Inf.** | **Inf.** | **Inf.** | **Inf.** | **Inf.** | **Inf.** | **Inf.** | **Inf.** | **Inf.** | **Inf.** | **Inf.** | **Inf.** | **Inf.** | **96.61** | **Inf.** | **Inf.** |
| F. St. Louis R. | **17.78** | 0.00 | *9.50* | *14.10* | **26.05** | --- | **116.45** | **Inf.** | **Inf.** | **Inf.** | **Inf.** | **Inf.** | **Inf.** | **Inf.** | **Inf.** | **Inf.** | **Inf.** | **Inf.** | **Inf.** | **Inf.** | **Inf.** | **Inf.** | *28.24* | **Inf.** | **Inf.** |
| G. L. Nipigon | **Inf.** | *11.21* | **Inf.** | **Inf.** | **Inf.** | *11.97* | --- | **Inf.** | **Inf.** | **Inf.** | **Inf.** | **Inf.** | **Inf.** | **Inf.** | **Inf.** | **Inf.** | **Inf.** | **Inf.** | **Inf.** | **Inf.** | **Inf.** | **Inf.** | **42.29** | **Inf.** | **Inf.** |
| H. Portage L. | **Inf.** | **Inf.** | **22.09** | **23.42** | **18.54** | **30.04** | **Inf.** | --- | **Inf.** | **Inf.** | **Inf.** | **Inf.** | **132.66** | **Inf.** | **Inf.** | **Inf.** | **Inf.** | **Inf.** | **Inf.** | **Inf.** | **Inf.** | **Inf.** | **40.13** | **Inf.** | **Inf.** |
| I. Muskegon R. | **27.44** | **Inf.** | *7.38* | **14.85** | 0.00 | **26.15** | **Inf.** | *14.48* | --- | **Inf.** | **54.23** | **Inf.** | **Inf.** | **Inf.** | **Inf.** | **Inf.** | **Inf.** | **Inf.** | **Inf.** | **Inf.** | **Inf.** | **Inf.** | **36.44** | **98.92** | **Inf.** |
| J. Thunder Bay | **Inf.** | **Inf.** | **16.05** | **19.56** | *12.90* | **Inf.** | **Inf.** | 0.61 | *8.74* | --- | **55.02** | **Inf.** | **Inf.** | **Inf.** | **Inf.** | **Inf.** | **Inf.** | **Inf.** | **Inf.** | **97.06** | **Inf.** | **Inf.** | **45.22** | **Inf.** | **Inf.** |
| K. Flint R. | **23.66** | **26.15** | *12.97* | *14.27* | *9.99* | **25.64** | **Inf.** | 3.60 | *6.21* | 1.80 | --- | **Inf.** | **65.64** | **Inf.** | **Inf.** | **Inf.** | **Inf.** | **Inf.** | **77.55** | **88.53** | **121.78** | **Inf.** | **33.72** | **94.90** | **Inf.** |
| L. Moon/Musquash R. | **Inf.** | **Inf.** | *7.40* | *14.79* | 1.43 | **29.02** | **Inf.** | *11.12* | 0.99 | *6.84* | *6.87* | --- | **Inf.** | **Inf.** | **Inf.** | **Inf.** | **Inf.** | **Inf.** | **Inf.** | **Inf.** | **Inf.** | **Inf.** | **54.72** | **Inf.** | **Inf.** |
| M. Thames R. | **Inf.** | **Inf.** | **27.27** | **27.11** | **24.19** | **Inf.** | **Inf.** | 2.19 | **16.62** | 3.74 | 2.69 | **17.76** | --- | 21.34 | 14.22 | *30.93* | **Inf.** | **Inf.** | **85.15** | **46.62** | **71.77** | **Inf.** | *26.11* | **52.83** | **Inf.** |
| N. Detroit R. | **Inf.** | **Inf.** | **Inf.** | **Inf.** | **Inf.** | **Inf.** | **Inf.** | 4.18 | **21.84** | *6.31* | *6.12* | **25.88** | 0.15 | --- | *25.07* | **69.76** | **Inf.** | **Inf.** | **Inf.** | **86.18** | **Inf.** | **Inf.** | **33.81** | **Inf.** | **Inf.** |
| O. W. basin L. Erie | **Inf.** | **Inf.** | **Inf.** | **Inf.** | **27.44** | **Inf.** | **Inf.** | 2.24 | **19.25** | 4.09 | 5.40 | **24.01** | 0.21 | 0.64 | --- | **64.88** | **Inf.** | **Inf.** | **Inf.** | **86.70** | **129.17** | **Inf.** | **33.64** | **Inf.** | **Inf.** |
| P. E. basin L. Erie | **Inf.** | **Inf.** | **Inf.** | **Inf.** | **Inf.** | **Inf.** | **Inf.** | 4.19 | **20.64** | *7.22* | *8.77* | **25.72** | 0.33 | *7.12* | 2.85 | --- | **Inf.** | **Inf.** | **Inf.** | **Inf.** | **Inf.** | **Inf.** | **33.25** | **Inf.** | **Inf.** |
| Q. Historic walleye | **Inf.** | **Inf.** | **Inf.** | **Inf.** | **Inf.** | **Inf.** | **Inf.** | **Inf.** | **Inf.** | **Inf.** | **25.80** | **Inf.** | **23.26** | **32.24** | **Inf.** | **Inf.** | --- | **63.03** | **Inf.** | **Inf.** | **Inf.** | **Inf.** | **82.92** | **Inf.** | **Inf.** |
| R. “Blue pike” | **Inf.** | **Inf.** | **Inf.** | **Inf.** | **Inf.** | **Inf.** | **Inf.** | **32.24** | **Inf.** | **Inf.** | **24.49** | **Inf.** | **23.26** | **Inf.** | **Inf.** | **Inf.** | 0.00 | --- | **Inf.** | **Inf.** | **Inf.** | **Inf.** | **123.09** | **Inf.** | **Inf.** |
| S. Pigeon L. | **Inf.** | **Inf.** | **Inf.** | **Inf.** | **Inf.** | **Inf.** | **Inf.** | **14.91** | **Inf.** | **17.95** | *13.16* | **Inf.** | *8.70* | **16.33** | **19.01** | **17.58** | 5.60 | *6.12* | --- | 22.62 | **122.84** | **Inf.** | *29.05* | **99.45** | **Inf.** |
| T. Bay of Quinte | **Inf.** | **Inf.** | **Inf.** | **Inf.** | **Inf.** | **Inf.** | **Inf.** | *10.25* | **Inf.** | *12.80* | *11.49* | **Inf.** | 5.40 | *12.00* | *13.04* | *10.87* | *9.39* | *9.88* | 0.39 | --- | **104.12** | **Inf.** | *31.10* | **69.83** | **Inf.** |
| U. Oneida L. | **Inf.** | **Inf.** | **Inf.** | **Inf.** | **Inf.** | **Inf.** | **Inf.** | **Inf.** | **Inf.** | **Inf.** | **30.04** | **Inf.** | **24.62** | **Inf.** | **Inf.** | **25.13** | **22.69** | **23.92** | **25.43** | **26.82** | --- | **Inf.** | **37.85** | **46.14** | **127.57** |
| V. L. Mistassini | **16.41** | 4.84 | 2.13 | *7.84* | *12.81* | 3.71 | **23.71** | **26.96** | *14.10* | **21.21** | **15.08** | *14.11* | **30.85** | **Inf.** | **Inf.** | **Inf.** | **Inf.** | **Inf.** | **Inf.** | **Inf.** | **Inf.** | --- | **113.79** | **Inf.** | **Inf.** |
| W. Ohio R. | **16.88** | **25.50** | **21.60** | **18.21** | **17.13** | **25.50** | **Inf.** | *9.35* | *14.14* | *10.10* | *8.16* | **18.56** | 5.73 | *9.76* | *6.92* | 2.98 | **29.02** | **27.44** | **17.11** | *12.56* | **15.70** | **23.11** | --- | *25.33* | **49.91** |
| X. New R. | **Inf.** | **Inf.** | **Inf.** | **Inf.** | **Inf.** | **Inf.** | **Inf.** | **Inf.** | **Inf.** | **Inf.** | **Inf.** | **Inf.** | **Inf.** | **Inf.** | **Inf.** | **Inf.** | **Inf.** | **Inf.** | **Inf.** | **Inf.** | **Inf.** | **Inf.** | *10.11* | --- | **Inf.** |
| Y. North R. | **20.34** | **22.14** | **22.47** | **21.01** | **21.71** | **23.68** | **22.43** | **23.37** | **21.18** | **21.48** | **20.81** | **25.01** | **20.91** | **27.84** | **26.35** | **20.46** | **22.00** | **17.20** | **20.59** | **20.04** | *14.49* | **24.01** | *8.37* | **18.28** | --- |
