# Supplementary material for: A population genetic window into the past and future of the walleye Sander vitreus: relation to historic walleye and the extinct “blue pike” S. v. “glaucus”
Source: BMC Evol Biol. 2014 Jun 17;14:133. doi: 10.1186/1471-2148-14-133 (PMC4229939; doi:10.1186/1471-2148-14-133)
Supplement: Additional file 5 — Genetic isolation by geographic distance comparison among 23 contemporary spawning groups of walleye. A mtDNA control region (y = 0.64x–3.67, R2 = 0.10, p < 0.001) and B seven nuclear μsat loci (y = 0.06x–0.29, R2 = 0.23, p = 0.005). Results from the seven loci are identical to those for nine loci (data not shown; see Stepien et al. [30]). Letters correspond to spawning group labels from Table 1. [file 1471-2148-14-133-S5.doc]

**Additional file 7**

**Geneclass2 [57] assignments among contemporary walleye spawning groups.** Values=percentage assignment, parentheses=number of individuals assigning to that group, **bold**=self-assignment, and *italics*=greatest assignment. Numbers in parentheses next to spawning group denote sample size.

| **Location** | **A.** | **B.** | **C.** | **D.** | **E.** | **F.** | **G.** | **H.** | **I.** | **J.** | **K.** | **L.** | **M.** | **N.** | **O.** | **P.** | **S.** | **T.** | **U.** | **V.** | **W.** | **X.** | **Y.** |
| --- | --- | --- | --- | --- | --- | --- | --- | --- | --- | --- | --- | --- | --- | --- | --- | --- | --- | --- | --- | --- | --- | --- | --- |
| A. Cedar L. (*N*=25) | **28 (7)** | - | *32 (8)* | - | - | 4( 1) | 4 (1) | 4 (1) | - | 8 (2) | 8 (2) | - | - | - | - | 4 (1) | - | - | 4 (1) | - | - | 4 (1) | - |
| B. L. Winnipeg (25) | 8 (2) | ***36 (9)*** | 20 (5) | - | - | 4 (1) | 4 (1) | - | 4 (1) | - | - | - | 8 (2) | - | - | 8 (2) | - | - | - | - | - | 8 (2) | - |
| C. L. of the Woods (30) | - | 3 (1) | ***53 (16)*** | - | - | 10 (3) | 7 (2) | 3 (1) | 3 (1) | - | 3 (1) | - | - | - | 7 (2) | 7 (2) | - | - | - | - | 3 (1) | - | - |
| D. McKim L. (25) | - | 4 (1) | 4 (1) | ***84 (21)*** | 4 (1) | - | - | - | - | - | - | - | - | - | - | 4 (1) | - | - | - | - | - | - | - |
| E. Mille Lacs (39) | - | - | 8 (3) | - | ***56 (22)*** | - | - | - | - | - | - | - | - | 15 (6) | 5 (2) | 8 (3) | - | 3 (1) | - | - | - | 5 (2) | - |
| F. St. Louis R. (28) | - | - | 4 (1) | - | - | ***43 (12)*** | - | - | - | 4 (1) | - | - | 4 (1) | 4 (1) | 18 (5) | 18 (5) | - | - | - | - | - | 4 (1) | - |
| G. L. Nipigon (30) | - | 7 (2) | *23 (7)* | - | - | 10 (3) | **17 (5)** | - | - | - | - | - | 7 (2) | 7 (2) | 7 (2) | 20 (6) | - | - | - | - | - | 3 (1) | - |
| H. Portage L. (56) | - | 2 (1) | - | - | - | 7 (4) | - | **14 (8)** | 4 (2) | - | 2 (1) | 4 (2) | 11 (6) | 5 (3) | 11 (6) | *25 (14)* | - | 4 (2) | - | - | 2 (1) | 11 (6) | - |
| I. Muskegon R. (50) | - | - | - | - | - | 6 (3) | - | - | ***54 (27)*** | - | 12 (6) | - | - | 6 (3) | 4 (2) | 6 (3) | - | 2 (1) | - | - | - | 8 (4) | - |
| J. Thunder Bay (40) | - | - | - | - | - | 3 (1) | - | - | 3 (1) | ***25 (10)*** | *25 (10)* | - | - | - | 23 (9) | 8 (3) | 3 (1) | 8 (3) | - | - | - | 5 (2) | - |
| K. Flint R. (44) | - | - | - | - | - | - | - | - | 27 (12) | 5 (2) | ***39 (17)*** | - | 5 (2) | 2 (1) | - | 9 (4) | 2 (1) | 5 (2) | - | - | - | 7 (3) | - |
| L. Moon/Musquash R. (35) | - | - | - | - | - | - | - | 6 (2) | 6 (2) | - | 6 (2) | ***51 (18)*** | - | 6 (2) | 17 (6) | 3 (1) | - | 3 (1) | - | - | - | 3 (1) | - |
| M. Thames R. (39) | - | - | 5 (2) | - | - | - | - | - | 8 (3) | - | 8 (3) | - | **5 (2)** | 21 (8) | 15 (6) | *31 (12)* | - | 3 (1) | - | - | - | 5 (2) | - |
| N. Detroit R. (123) | - | - | - | - | - | 2 (2) | - | - | 2 (2) | - | 2 (3) | 1 (1) | 10 (12) | **11 (13)** | 25 (31) | *38 (47)* | 3 (4) | 1 (1) | - | - | 1 (1) | 5 (6) | - |
| O. W. basin L. Erie (211) | - | - | - | - | - | 1 (2) | - | - | 1 (2) | - | 3 (7) | - | 11 (24) | 15 (31) | **23 (48)** | *37 (78)* | 1 (2) | 3 (7) | - | - | - | 5 (10) | - |
| P. E. basin L. Erie (137) | - | - | - | - | - | 2 (3) | - | - | 3 (4) | - | 1 (2) | - | 7 (9) | 7 (9) | 17 (23) | ***57 (78)*** | 2 (3) | - | - | - | - | 4 (6) | - |
| S. Pigeon L. (29) | - | - | - | - | - | - | - | - | - | - | 17 (5) | - | 3 (1) | 3 (1) | - | 10 (3) | **14 (4)** | *48 (14)* | - | - | - | 3 (1) | - |
| T. Bay of Quinte (50) | - | - | - | - | - | - | - | - | - | 2 (1) | 2 (1) | - | 10 (5) | 6 (3) | 10 (5) | 24 (12) | 10 (5) | ***30 (15)*** | - | - | - | 6 (3) | - |
| U. Oneida L. (25) | - | - | - | - | - | - | - | - | 4 (1) | - | - | - | 16 (4) | - | 4 (1) | 8 (2) | - | 4 (1) | **24 (6)** | - | - | *40 (10)* | - |
| V. L. Mistassini (40) | - | - | - | - | - | 5 (2) | - | - | - | - | - | - | - | 3 (1) | 5 (2) | 15 (6) | - | - | 3 (1) | ***58 (23)*** | - | 13 (5) | - |
| W. Ohio R. (4) | - | - | - | - | - | *25 (1)* | - | - | - | - | - | - | - | - | *25 (1)* | - | - | *25 (1)* | - | - | **-** | *25 (1)* | - |
| X. New R. (35) | - | - | - | - | - | 3 (1) | - | - | 6 (2) | - | 9 (3) | - | 6 (2) | 3 (1) | 9 (3) | 20 (7) | - | 6 (2) | 3 (1) | - | - | ***37 (13)*** | - |
| Y. North R. (5) | - | - | - | - | - | - | - | - | - | - | - | - | - | - | - | - | - | - | - | - | - | - | ***60 (3)*** |
