# Supplementary material for: A population genetic window into the past and future of the walleye Sander vitreus: relation to historic walleye and the extinct “blue pike” S. v. “glaucus”
Source: BMC Evol Biol. 2014 Jun 17;14:133. doi: 10.1186/1471-2148-14-133 (PMC4229939; doi:10.1186/1471-2148-14-133)
Supplement: Additional file 6 — Pairwise exact tests of genetic differentiation among population samples (lettered) for control region sequence data (below diagonal) and seven nuclear μsat loci (above diagonal). Results are congruent to FST comparisons and those from the seven nuclear μsat loci data are identical to values calculated based on nine loci (data not shown; also see Stepien et al. [30]), with difference at just the thousandth decimal place. Inf. = Infinite value denoted by Genepop [119], bold = significant following sequential Bonferroni corrections, italics = significant at α = 0.05, and normal text = not significant. [file 1471-2148-14-133-S6.doc]

**
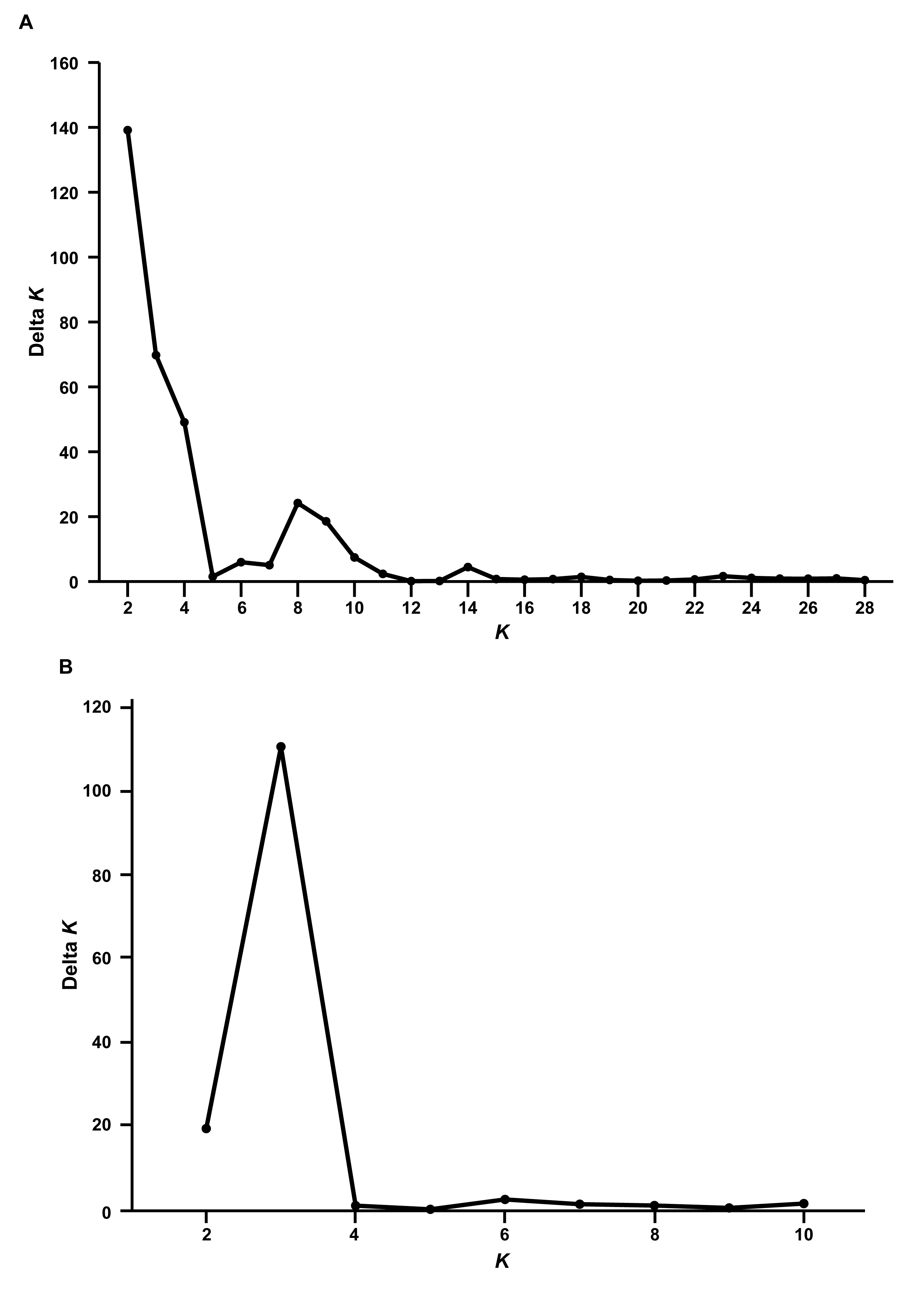
**

**Additional file 8**

**Graph of *K* vs. *ΔK* based on Evanno et al. [123], showing the optimal *K* A contemporary walleye spawning groups and B contemporary Lakes Erie and Ontario and historic Lake Erie walleye (with “blue pike”).**
