# Supplementary material for: A population genetic window into the past and future of the walleye Sander vitreus: relation to historic walleye and the extinct “blue pike” S. v. “glaucus”
Source: BMC Evol Biol. 2014 Jun 17;14:133. doi: 10.1186/1471-2148-14-133 (PMC4229939; doi:10.1186/1471-2148-14-133)
Supplement: Additional file 7 — Geneclass2 [[59]] assignments among contemporary walleye spawning groups. Values = percentage assignment, parentheses = number of individuals assigning to that group, bold = self-assignment, and italics = greatest assignment. Numbers in parentheses next to spawning group denote sample size. [file 1471-2148-14-133-S7.doc]

**
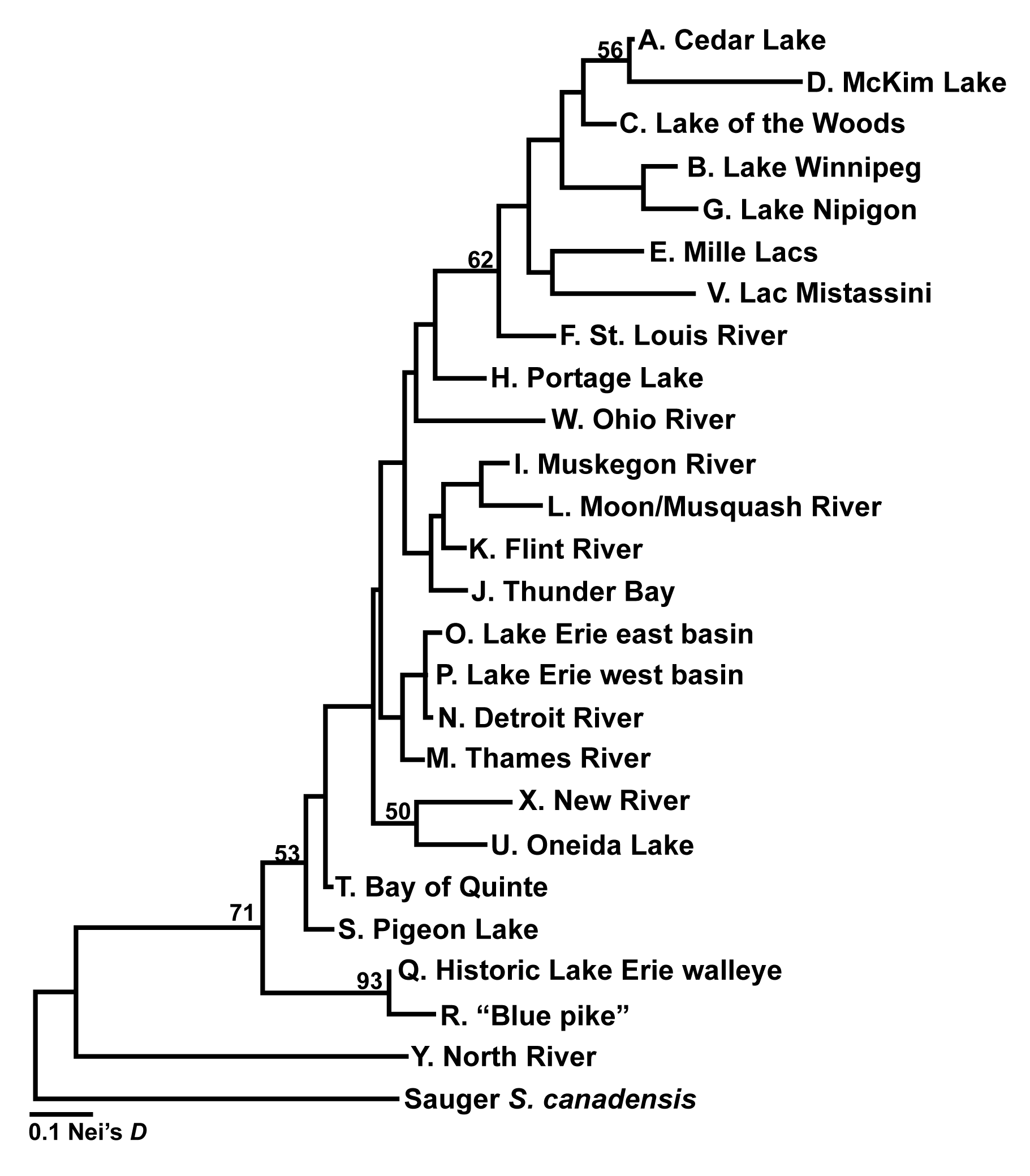
**

**Additional file 9**

**Population genetic distance neighbor joining tree for contemporary walleye *Sander vitreus vitreus* spawning groups, historic walleye, and “blue pike” *S. v. “glaucus”* in relation to sauger *S. canadensis*.** Tree is based on combined frequencies of mtDNA control region haplotypes and alleles from the seven nuclear μsat loci.
