# Supplementary material for: A population genetic window into the past and future of the walleye Sander vitreus: relation to historic walleye and the extinct “blue pike” S. v. “glaucus”
Source: BMC Evol Biol. 2014 Jun 17;14:133. doi: 10.1186/1471-2148-14-133 (PMC4229939; doi:10.1186/1471-2148-14-133)
Supplement: Additional file 8 — Graph of K vs. ΔK based on Evanno et al. [[123]], showing the optimal K A contemporary walleye spawning groups and B contemporary Lakes Erie and Ontario and historic Lake Erie walleye (with “blue pike”). [file 1471-2148-14-133-S8.doc]

**Additional file 10**

**Latitude (Lat.) and longitude (Long.) for each contemporary walleye spawning group used in analyses.** Letters correspond to locations from Figure 1 and Table 1.

| **Location** | **Lat.** | **Long.** |
| --- | --- | --- |
| A. Cedar L. | 53.3300 | -100.1000 |
| B. L. Winnipeg | 52.7388 | -97.8628 |
| C. L. of the Woods | 49.0367 | -94.9272 |
| D. McKim L. | 50.8669 | -92.8031 |
| E. Mille Lacs | 46.2326 | -93.6477 |
| F. St. Louis R. | 46.6679 | -92.2889 |
| G. L. Nipigon | 49.7237 | -88.6145 |
| H. Portage L. | 47.0225 | -88.5097 |
| I. Muskegon R. | 43.4158 | -85.8087 |
| J. Thunder Bay | 45.0200 | -83.4300 |
| K. Flint R. | 43.3300 | -84.0543 |
| L. Moon/Musquash R. | 44.9594 | -79.8811 |
| M. Thames R. | 42.3171 | -82.4363 |
| N. Detroit R. | --- | --- |
| N1. Belle Isle | 42.3469 | -82.9535 |
| N2. Fighting Is. | 42.2378 | -83.1295 |
| N3. Grosse Ile | 42.1177 | -83.1781 |
| O. Western L. Erie | --- | --- |
| O1. Huron R. | 42.0899 | -83.2902 |
| O2. Hen Is. | 41.8024 | -82.7804 |
| O3. Maumee R. | 41.5594 | -83.6492 |
| O4. Sandusky R. | 41.3421 | -83.1091 |
| P. Eastern L. Erie | --- | --- |
| P1. Van Buren Bay | 42.4600 | -79.4100 |
| P2. Cattaraugus Ck. | 42.5684 | -79.1041 |
| S. Pigeon L. | 44.4703 | -78.4942 |
| T. Bay of Quinte | 44.0671 | -77.0719 |
| U. Oneida L. | 43.2800 | -75.4400 |
| V. Lac Mistassini | 50.9500 | -73.7000 |
| W. Ohio R. | 39.6675 | -80.8641 |
| X. New R. | 36.7109 | -80.9589 |
| Y. North R. | 33.3264 | -87.5333 |
