# Supplementary material for: A population genetic window into the past and future of the walleye Sander vitreus: relation to historic walleye and the extinct “blue pike” S. v. “glaucus”
Source: BMC Evol Biol. 2014 Jun 17;14:133. doi: 10.1186/1471-2148-14-133 (PMC4229939; doi:10.1186/1471-2148-14-133)
Supplement: Additional file 9 — Population genetic distance neighbor joining tree for contemporary walleye Sander vitreus vitreus spawning groups, historic walleye, and “blue pike” S. v. “glaucus” in relation to sauger S. canadensis. Tree is based on combined frequencies of mtDNA control region haplotypes and alleles from the seven nuclear μsat loci. [file 1471-2148-14-133-S9.doc]

**Additional file 11**

**Materials examined, including A museum specimens and B additional contemporary specimens that augmented prior work reported by Stepien et al. (2009, 2010, 2012).** Institutional abbreviations follow Leviton et al. [139]. Sample information is listed as follows: museum – lot number, number of samples, size range (SL, mm), locality, sampling date, and collector. Individuals that also were analyzed with molecular data are listed in the “Molecular Samples” section with their respective lot numbers and GenBank accession numbers. Individuals analyzed solely for molecular data are listed under “Molecular Samples” with lot number, number of samples, locality, sampling date, collector, and GenBank accession number.

*Sander vitreus “glaucus”* (Hubbs, 1926)

Morphological Samples:

UMMZ – 72090, 26, 202–270 mm SL, Lake Erie – near Erie PA, 30 Nov. 1923, Lay Bros. Fishing Co., paratypes identified by C.L. Hubbs.

USNM – 88458, 17, 204–345 mm SL, Lake Erie – near Erie PA, 1910, W.H. Kendall; 88459, 5, 265–330 mm SL, Lake Erie – near Erie PA, 1910, W.H. Kendall; 117481, 5, 230–270 mm SL, Lake Erie – near Erie PA, 1924, Keystone Fish Co., paratypes identified by C.L. Hubbs.

Molecular Samples:

For unique samples all information is listed. Samples also analyzed for morphology have complete information in the morphological section.

UMMZ – 72091, 22, Lake Erie – near Erie PA, 30 Nov. 1923, Lay Bros. Fishing Co., paratypes identified by C.L. Hubbs, GenBank:JX442948.

*Sander vitreus vitreus* (Mitchill, 1818).

Morphological Samples:

UMMZ – 55299, 1, 277 mm SL, Lake Erie – near Monroe MI, 30 Nov. 1919, C.L. Hubbs; 55456, 1, 214 mm SL, Lake Erie – near Monroe MI, 31 Oct. 1920, C.L. Hubbs; 72078, 1, 270 mm SL, Lake Erie – near Port Clinton OH, 30 Nov. 1923, Lay Bros. Fishing Co.; 72079, 1, 277 mm SL, Lake Erie – near Port Clinton OH, 30 Nov. 1923, Lay Bros. Fishing Co.; 72080, 1, 252 mm SL, Lake Erie – near Port Clinton OH, 30 Nov. 1923, Lay Bros. Fishing Co.; 72081, 1, 273 mm SL, Lake Erie – near Port Clinton OH, 30 Nov. 1923, Lay Bros. Fishing Co.; 72082, 1, 262 mm SL, Lake Erie – near Port Clinton OH, 30 Nov. 1923, Lay Bros. Fishing Co.; 72083, 1, 283 mm SL, Lake Erie – near Port Clinton OH, 30 Nov. 1923, Lay Bros. Fishing Co.; 72084, 1, 249 mm SL, Lake Erie – near Port Clinton OH, 30 Nov. 1923, Lay Bros. Fishing Co.; 72085, 1, 292 mm SL, Lake Erie – near Port Clinton OH, 30 Nov. 1923, Lay Bros. Fishing Co.; 72086, 1, 266 mm SL, Lake Erie – near Port Clinton OH, 30 Nov. 1923, Lay Bros. Fishing Co.; 72087, 1, 270 mm SL, Lake Erie – near Port Clinton OH, 30 Nov. 1923, Lay Bros. Fishing Co.; 72088, 1, 255 mm SL, Lake Erie – near Port Clinton OH, 30 Nov. 1923, Lay Bros. Fishing Co.; 72089, 1, 324 mm SL, Lake Erie – near Port Clinton OH, 30 Nov. 1923, Lay Bros. Fishing Co.; 72095, 1, 242 mm SL, Lake Erie – near Sandusky OH, 30 Nov. 1923, Lay Bros. Fishing Co.; 72096, 1, 198 mm SL, Lake Erie – near Sandusky OH, 30 Nov. 1923, Lay Bros. Fishing Co.; 133669, 3, 187–247 mm SL, Lake Erie – near Erie PA, 5 Jun. 1941, F.C. Ralph; 218003, 2, 203–236 mm SL, Maumee River – near Toledo OH, 25 Aug. 1893, P.H. Kirsch.

USNM – 9391, 1, 315 mm SL, Ecorse MI, 1872, G. Clarke; 12274, 1, 257 mm SL, Ohio River – near Cincinnati OH, 1890s, Milner; 12276, 1, 312 mm SL, Ohio River – near Cincinnati OH, 1890s, Milner; 22494, 1, 303 mm SL, Susquehanna River – near Port Deposit MD, 7 Apr. 1879, R.E. McClenahan; 23226, 1, 245 mm SL, Lake Champlain, unknown, unknown; 26261, 1, 194 mm SL, Wetumpka AL, 1881, J. Skinner; 34716, 1, 279 mm SL, Lake Erie – near Washington Market OH, 1884, H.L. Todd; 34828, 1, 202 mm SL, Hudson Bay CAN, 1884, W. Haydon; 43910, 1, 302 mm SL, Fort Snelling MN, 1890, E.A. Mearns; 63801, 1, 200 mm SL, Pitmans Creek – near Greenburg KY, 1909, A.J. Woolman; 68935, 1, 250 mm SL, Lake Huron – near Point Sanilac MI, 20 Jun. 1894, J.T. Scovell; 88446, 1, 340 mm SL, Fairport IA, early 1900s, T. Surber; 88454, 1, 348 mm SL, Fairport IA, early 1900s, T. Surber; 88456, 1, 385 mm SL, Fairport IA, early 1900s, T. Surber; 91473, 1, 198 mm SL, Newman’s Pond MD, 26 Nov. 1915, United States Bureau of Fisheries; 91474, 1, 209 mm SL, Newman’s Pond MD, 26 Nov. 1915, United States Bureau of Fisheries; 91475, 1, 199 mm SL, Newman’s Pond MD, 26 Nov. 1915, United States Bureau of Fisheries; 91476, 1, 209 mm SL, Newman’s Pond MD, 26 Nov. 1915, United States Bureau of Fisheries; 131332, 9, 272–338 mm SL, Lake of the Woods ON CAN, 1908, S. Meek; 125067, 1, 197 mm SL, Lake Erie – near Toledo OH, 12 Aug. 1894, United States Bureau of Fisheries; 154817, 1, 240 mm SL, Cromwell CN, 1952, H. Woodward; 154823, 1, 222 mm SL, Cromwell CN, 1952, L. Taylor.

Molecular Samples:

UMMZ – 55299, 72080-81, 72085-86, 72095, GenBank:JX442948; 72083, GenBank:KF954735; 243215, 1, Lake Erie – near Chelerna Isl. MI, 16 Aug. 1935, Rodeheffer, GenBank:JX442948; 243259, 4, Lake Erie – near Sturgeon Bar, MI, 16 Aug. 1935, Rodeheffer, GenBank:JX442948.

USNM – 69687, 1, Red Brook – near Harbor OH, 1 Aug. 1893, A.J. Woolman, GenBank:JX442948.

OSUM – 1449, 1, Lake Erie – near Stone’s Cove, Jun. 1939, M.B. Trautman, GenBank:JX442948; 4229, 6, Lake Erie – near Peach Point, 7 Feb. 1941, K.H. Doan, GenBank:JX442948; 4232, 2, Lake Erie – near Put–in–Bay OH, 12 Feb. 1941, K.H. Doan, GenBank:JX442948; 5721, 1, Lake Erie – near Put–in–Bay OH, 30 Oct. 1942, K.H. Doan, GenBank:JX442948; 6073, 2, Lake Erie – near Put–in–Bay OH, 5 Oct. 1943, K.H. Doan, GenBank:JX442948, 16431, 3, Sandusky Bay Lake Erie, 17 Aug. 1939, M.B. Trautman, GenBank:JX442948; 16467, 6, Sandusky Bay Lake Erie, 17 Aug. 1939, M.B. Trautman, GenBank:JX442948.

**B Description of increases in sample sites and sizes** for the microsatellite dataset from this study relative to the original Stepien et al. [28] database. Letter codes match those from Table 1 and Figure 1.

Sample sizes were increased for sampling sites at Cedar Lake (site A by +9 individuals), McKim Lake (D, +5), Thames River (M, +1), Detroit River (N, +83), Huron River (O1, +20), and Oneida Lake (U, +5). We added six new spawning sites (+181 individuals) to increase coverage across the Canadian Shield, including Lake Winnipeg (B), Lake of the Woods (C), Lake Nipigon (G), Portage Lake (H), Pigeon Lake (S), and Lac Mistassini (V; Figure 1, Table 1). The Ohio River (W) sample here contained just four individuals remaining from earlier study [40]
